# Cooperative Free Energy: Induced Protein–Protein Interactions and Cooperative Solvation in Ternary Complexes

**DOI:** 10.1021/acs.jctc.5c00736

**Published:** 2025-08-20

**Authors:** Shu-Yu Chen, Riccardo Solazzo, Marianne Fouché, Hans-Jörg Roth, Birger Dittrich, Sereina Riniker

**Affiliations:** † Department of Chemistry and Applied Biosciences, ETH Zurich, Vladimir-Prelog-Weg 2, Zurich 8093, Switzerland; ‡ 27219 Novartis Biomedical Research, Novartis Campus, Basel 4002, Switzerland

## Abstract

Protein–protein interactions (PPIs) play an essential role in biological processes. Molecules that stabilize or induce PPIs in ternary complexes have received growing attention for their therapeutic potential in engaging “undruggable” targets and their high selectivity. Here, we investigate the thermodynamics of the cooperative phenomenon in ternary complexes. The thermodynamics of cooperativity are characterized by the cooperative free energy, which comprises induced PPIs, cooperative solvation free energy, ligand-associated geometric free-energy costs, and gas-phase correlation. Importantly, the induced PPIs only account for the binding affinity between stabilized conformations of the protein partners, i.e., the free-energy change associated with the conformational transition during protein–ligand binding is not accounted for. By introducing an approximated expression for the cooperative free energy, we developed a rapid computational method, which allowed us to crudely predict cooperativity in eight ternary complexes (Kendall τ = 0.79). We highlight that the term cooperativity used in protein–protein stabilization does not represent the cooperativity phenomenon in three-body systems. We also critically discuss the counterintuitive interpretation of cooperative free energy due to its asymmetric nature. Our study shows how cooperativity stabilizes ternary complexes and provides a thermodynamic basis of cooperativity in protein–ligand–protein complexes.

## Graphical abstract

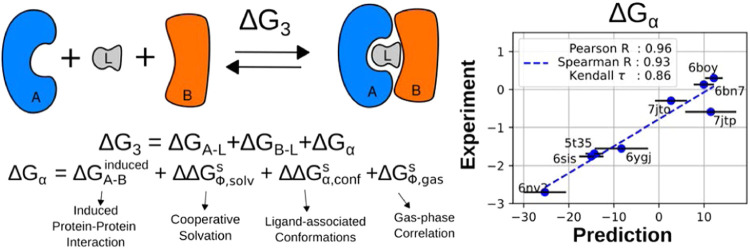

## Introduction

Protein–protein interactions (PPIs) play a substantial role in essential biological processes.^1,2^ Modulating protein functions or inducing targeted protein degradation using PPI stabilizers has become a promising therapeutic strategy, which covers a broad range of physiological processes such as degradation, inhibition, stimulation, and translocation.^3–11^ Of particular interest are proteolysis-targeting chimeras (PROTACs) consisting of a linker between an E3 ligase-binding warhead and a target-binding warhead to facilitate ubiquitination and subsequent degradation of the targeted protein.^12–18^ According to the mathematical models for noninteracting proteins, the equilibrium concentration of the ternary complex can be analytically determined given the initial concentration of each species (i.e., the two protein partners and the ligand), the dissociation constants of the two protein–ligand pairs, and the intrinsic cooperativity (of the ligand, denoted as α).^19,20^ In a noncooperative system, the ternary complex forming species must possess some binding affinity to both proteins to induce the formation of a ternary complex, termed the dual-binding mechanism.^21^ Systems that do not follow the dual-binding mechanism can still form ternary complexes through high cooperativity, including allosteric modulation.^22–24^ Structural determinations and mutagenesis studies have shown that cooperativity is directly related to the induced *de novo* protein–protein contacts and is orthogonal to the binary protein–ligand binding affinities.^13,25^ PROTACs can hence induce favorable or unfavorable PPIs to exhibit positive (α > 1) or negative (0 < α < 1) cooperativity through different scaffolds of the linker, respectively. Although a noncooperative (α = 1) ternary complex can be stabilized by the dual-binding mechanism, it induces the hook effect (also knoa high ligand dosage as seen for bifunctional PPI stabilizers.^97^ The hook effect starts to become disruptive for the formation of ternary complexes around compound concentrations that are one logarithmic unit over the corresponding ternary *K*_d_ values due to the overwhelming concentration of the binary complexes of ligands and proteins.^26–28^ In contrast, high cooperativity mitigates the hook effect, which implies a wider working window of ligand concentration while maintaining high concentration of the ternary complex.^98^ The dual-binding mechanism requirement and the partner-specific induced PPIs make PPI stabilizers highly selective and attractive therapeutic alternatives.^13,23,25,28–30^

In mathematics and physics, the cooperative phenomenon describes the emergent properties in a many-body system that are not expected from the separate pairwise behaviors. In chemistry and biology, the term cooperativity is often used to describe the protein-folding process and the allosteric modulation of enzymes upon ligand binding.^31–37^ Historically, cooperativity in PPI stabilization is an asymmetric quantity and usually refers to the cooperativity of the ligand, i.e., describing how the binding between a ligand and the first protein is facilitated by the presence of the second protein,^38^ although other definitions have also been used.^39,40^ Experimentally, absolute (or intrinsic) cooperativity, denoted by the ratio between the dissociation constants *K*_d_ of the binary complex and the ternary complex, is difficult to measure because adding the second protein into a solution enriches not only the ternary complex but also the new protein–protein and protein–ligand binary complexes. In contrast, apparent cooperativity is usually measured by monitoring the shift in the EC_50_ values induced by the presence of the second protein at a saturating concentration during titration experiments. Mathematical models are required to derive the absolute cooperativity (shift in the dissociation constant, *K*_d_, independent of species concentration) from the apparent cooperativity (shift in EC_50_, dependent on species concentration).^41^ Besides a qualitative correlation between induced PPIs and cooperativity,^13,25^ the exact physical interpretation of cooperativity in ternary complexes at a molecular level often remains elusive, making rational optimization of cooperativity in drug design challenging.

In the present study, we first revisit how cooperativity modulates the concentration of a ternary complex in equilibrium using enzyme kinetics and distinguish roles of binary protein–ligand binding affinities from cooperativity. We show that the hook effect is a direct consequence of strong binary binding, which can be alleviated by weakening the corresponding protein–ligand interaction and increasing cooperativity. To eliminate the asymmetry of cooperativity, we introduce the concept of *reduced cooperativity*, which captures only the nonpairwise effects of the three-body system. Under the assumption of a rigid ligand and two independent ligand-binding events in the gas-phase, cooperativity can be approximated as the sum of the induced PPIs and the cooperative solvation free energy. Both quantities can be estimated from molecular dynamics (MD) simulations. Lastly, different types of induced PPIs caused by geometric perturbations are discussed, and the protein–protein geometries resolved in the crystal structures are compared.

## Results and Discussion

### Modulation of Ternary Complexation with Cooperativity in Systems with Weak Protein–Protein Interactions

We consider a system consisting of two weakly interacting proteins *A* and *B*. When adding ligand *L*, which interacts with both *A* and *B*, the formation of the binary complexes *AL* and *BL* as well as the ternary complex *ALB* is observed. At equilibrium, the relative concentrations between species can be expressed in terms of dissociation constants *K*_*A*–*L*_, *K*_*B*–*L*_, *K*_*AL*–*B*_, and *K*_*BL*–*A*_, where $K_{X\text{−}Y}=\frac{\left[X\right]\left[Y\right]}{\left[\mathrm{XY}\right]}$ for *X*, *Y* ∈ {*A*, *B*, *L*} and [X] denotes the equilibrium concentration of species *X*. As depicted in Figure 1A, the ternary complex *ALB* can be formed by two consecutive binding pathways. An effective ternary dissociation constant *K*_3_ can be described by the ratio between the individual molecules and the ternary complex

1 $$K_{3}=\frac{\left[A\right]\left[L\right]\left[B\right]}{\left[\mathrm{ALB}\right]}=K_{A-L}K_{\mathrm{AL}-B}=K_{B-L}K_{A-\mathrm{BL}}=\alpha^{-1}K_{A-L}K_{B-L}$$

where the cooperativity α is a dimensionless quantity defined as

2 $$\alpha =\frac{\left[\mathrm{AL}\right]\left[\mathrm{BL}\right]}{\left[\mathrm{ABL}\right]\left[L\right]}=\frac{K_{A-L}}{K_{A-\mathrm{BL}}}=\frac{K_{B-L}}{K_{\mathrm{AL}-B}}$$

The ratio $\frac{K_{A-L}}{K_{A-\mathrm{BL}}}$ signifies how protein *B* facilitates binding of ligand *L* to protein *A*. When *K*_*A*–*BL*_ > *K*_*A*–*L*_ (α < 1, i.e., negative cooperativity), the presence of protein *B* impedes binding, whereas when *K*_*A*–*BL*_ = *K*_*A*–*L*_ (α = 1, i.e., no cooperativity), protein *B* has no impact. On the other hand, when *K*_*A*–*BL*_ < *K*_*A*–*L*_ (α > 1, i.e., positive cooperativity), protein *B* facilitates binding. The maximal concentration of a ternary complex is reached when the most limiting molecule present is completely aggregated in ternary form, i.e.

3 $${\left[\mathrm{ALB}\right]}^{\max}=\min\left\{\left[A_{\mathrm{tot}}\right],\left[B_{\mathrm{tot}}\right],\left[L_{\mathrm{tot}}\right]\right\}$$

with which we define the fraction of ternary-complex formation *f*_3_ = [*ALB*]/[*ALB*]^max^ ≤ 1.

**Figure 1:**
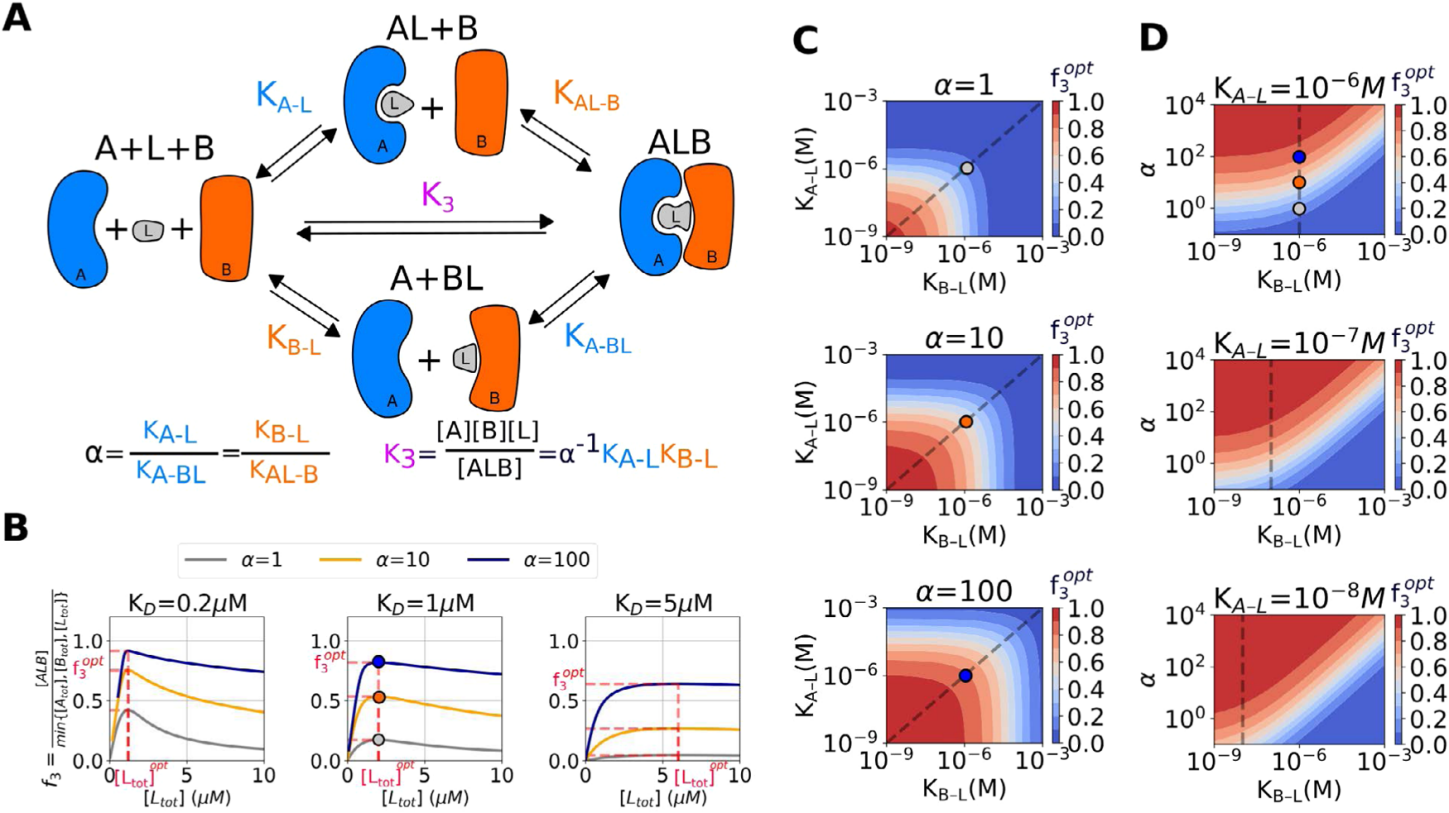
Ternary complex formation between proteins *A*, *B*, and ligand *L*. (A): Pathways from molecules *A*, *B*, and *L* to binary complexes *AL* and *BL*, and to ternary complex *ALB* with dissociation constants *K*_*A*–*L*_, *K*_*B*–*L*_, *K*_*AL*–*B*_, and *K*_*A*–*BL*_ and cooperativity α. (B): Fraction of ternary-complex formation *f*_3_ as a function of total ligand concentration with different cooperativity values α (gray: α = 1, orange: α = 10, blue: α = 100) under even binding condition, i.e., *K*_*A*–*L*_ = *K*_*B*–*L*_ = *K*_*d*_. The optimal [*L*_tot_] and the corresponding *f*_3_^opt^ are highlighted in red dashed lines. (C): Response of the optimal fraction *f*_3_^opt^ against the dissociation constants *K*_*A*–*L*_ and *K*_*B*–*L*_ with a fixed α. (D): Response of the optimal fraction *f*_3_^opt^ against the dissociation constant *K*_*B*–*L*_ and cooperativity α with a fixed dissociation constant *K*_*A*–*L*_. In (B–D), [*A*_tot_] and [*B*_tot_] are set at 1 μM and the condition of *f*_3_^opt^ as *K*_*A*–*L*_ = *K*_*B*–*L*_ = 1 μM at different α are shown in circles. The gray dashed lines in (C, D) indicate the condition of even binding, i.e., *K*_*A*–*L*_ = *K*_*B*–*L*_.

Following the work by Yang and Hlavacek,^20^ the concentration of the ternary complex at equilibrium can be obtained using the mass conservation law and the dissociation constants (see Section S1 in the Supporting Information for the derivation), given the initial concentrations of each molecule [*A*_tot_], [*B*_tot_], and [*L*_tot_]­

4 $$\left[\mathrm{ALB}\right]=\frac{C-\sqrt{C^{2}-4\left[A_{\mathrm{tot}}\right]\left[B_{\mathrm{tot}}\right]}}{2}$$

with

5 $$C\equiv \left[A_{\mathrm{tot}}\right]+\left[B_{\mathrm{tot}}\right]+\frac{\left(\left[L\right]+K_{A-L}\right)\left(\left[L\right]+K_{B-L}\right)}{\alpha \left[L\right]}$$

and the total concentration of the ligand being

6 $$\left[L_{\mathrm{tot}}\right]=\left[L\right]+\frac{\left[L\right]\left[A_{\mathrm{tot}}\right]}{\left[L\right]+K_{A-L}}+\frac{\left[L\right]\left[B_{\mathrm{tot}}\right]}{\left[L\right]+K_{B-L}}+\left(1-\frac{\left[L\right]}{\left[L\right]+K_{A-L}}+\frac{\left[L\right]}{\left[L\right]+K_{B-L}}\right)\left[\mathrm{ALB}\right]$$

Figure 1B shows the response of ternary-complex concentration [*ALB*] as a function of [*L*_tot_] with varying cooperativities and binary binding affinities according to eqs 4 and 6. Figure 1B also shows that the concentration [*ALB*] peaks at a certain total ligand concentration, which we denote as the optimal concentrations in the present study. The optimal ternary-complex concentration [*ALB*]^opt^ occurs at an optimal total ligand concentration [*L*_tot_]^opt^ with a corresponding monomer concentration [*L*]^opt^ (see Section S1 in the Supporting Information for the derivation)

7 $${\left[L_{\mathrm{tot}}\right]}^{\mathrm{opt}}=\sqrt{K_{A-L}K_{B-L}}+\frac{\left[A_{\mathrm{tot}}\right]}{1+\sqrt{K_{A-L}/K_{B-L}}}+\frac{\left[B_{\mathrm{tot}}\right]}{1+\sqrt{K_{B-L}/K_{A-L}}}$$

8 $${\left[L\right]}^{\mathrm{opt}}=\sqrt{K_{A-L}K_{B-L}}$$

Equation 7 indicates that the optimal total ligand concentration [*L*_tot_]^opt^ is determined by the two binary binding affinities instead of cooperativity (Figure 1B). Substituting eq 8 in eq 4 provides a scheme to obtain the optimal fraction $f_{3}^{\mathrm{opt}}=\frac{{\left[\mathrm{ALB}\right]}^{\mathrm{opt}}}{{\left[\mathrm{ALB}\right]}^{\max}}$ . These analytical expressions provide a way to monitor how α, *K*_*A*–*L*_, and *K*_*B*–*L*_ collectively modulate the magnitude of *f*_3_ and *f*_3_^opt^.

In the case of noncooperative strong binding (α = 1 and K_*A*–*L*_ = K_*B*–*L*_ = 0.2 μM, the gray line in the left panel of Figure 1B), a high concentration of [*L*_tot_] can lead to a strong hook effect (or Prozone phenomenon),^42^ indicated by the drop in *f*_3_ when [*L*_tot_] exceeds [*L*_tot_]^opt^. This is due to the overwhelming binary concentrations [*AL*] and [*BL*], often observed in bifunctional PPI stabilizers.^26^ The hook effect is mitigated when cooperativity increases (yellow and blue lines) or binding affinities decrease (middle and right panels of Figure 1B), which is indicated by the smoother decrease in *f*_3_ when [*L*_tot_] exceeds [*L*_tot_]^opt^.

Figure 1C,D depict the two-dimensional (2D) projection of *f*_3_^opt^ with a fixed cooperativity α and a fixed *K*_*A*–*L*_, respectively. Generally, *f*_3_^opt^ benefits the least from enhancing binding affinity between ligand and the stronger binding protein. When binding affinities between ligand and the two proteins are highly skewed (i.e., $\frac{K_{A-L}}{K_{B-L}}>2$ ), *f*_3_^opt^ can only be improved by enhancing the ligand-binding affinity of the weaker binding protein or by increasing cooperativity.

In summary, while the same *f*_3_^opt^ can be achieved through different combinations of *K*_*A*–*L*_, *K*_*B*–*L*_, and α (e.g., contour lines in Figure 1C,D), higher cooperativity α with weaker binding affinities can reach an equally high *f*_3_^opt^ with a milder hook effect (Figures 1B and S1 in the Supporting Information), making cooperativity an attractive optimization target even at the cost of a weakened binding affinity between the ligand and the proteins.

### Cooperative Free Energy

In an equilibrated isothermal–isobaric (NPT) system, the binary dissociation constant can be thermodynamically expressed as the difference in Gibbs free energy between the binary state (*G*_*AB*_) and the unary states (*G*_*A*_ and *G*_*B*_)­

9 $$K_{A-B}=c_{0}e^{\beta \left[G_{\mathrm{AB}}-\left(G_{A}+G_{B}\right)\right]}=c_{0}e^{\beta \Delta G_{A-B}}$$

where β^–1^ = k_*B*_ *T* is the product of the Boltzmann constant (*k*_B_) and temperature (*T*), *c*_0_ is the standard state concentration (1 molar), and Δ*G*_*A*–*B*_ is the binding free energy between proteins *A* and *B*. Similarly, the thermodynamic quantity of the effective ternary dissociation constant *K*_3_ in eq 1 describing the complexation from individual partners to the ternary complex can be expressed as

10 $$\Delta G_{3}=\beta^{-1}\log\frac{K_{3}}{c_{0}^{2}}=\beta^{-1}\log\left(\frac{K_{A-L}K_{B-L}}{c_{0}^{2}}\alpha^{-1}\right)=\Delta G_{A-L}+\Delta G_{B-L}+\Delta G_{\alpha }$$

with Δ*G*_α_ being the cooperative free energy defined as

11 $$\Delta G_{\alpha }=-\beta^{-1}\ln\alpha =\Delta G_{3}-\left(\Delta G_{B-L}+\Delta G_{A-L}\right)=G_{\mathrm{ALB}}+G_{L}-G_{\mathrm{AL}}-G_{\mathrm{BL}}$$

To remove the asymmetric nature of cooperative free energy, it is useful to introduce the *reduced cooperativity* ϕ with an associated free energy,

ΔGϕ=ΔGα−ΔGA−B=ΔG3−(ΔGA−B+ΔGB−L+ΔGA−L)=GALB−(GAB+GAL+GBL)+(GA+GB+GL)=ΔGAB−L−(ΔGA−L+ΔGB−L) 12

where Δ*G*_*AB*–*L*_ is the binding free energy between ligand and protein complex *AB*. The last line of eq 12 indicates that Δ*G*_ϕ_ quantifies the nonadditive part in addition to pairwise binding free energies. Rearranging eq 12 with the thermodynamic cycle depicted in Figure 2A, Δ*G*_ϕ_ can be decomposed into contributions from geometry (Δ*G*_ϕ,geo_^*s*^), cooperative solvation (Δ*G*_ϕ,solv_^s^), and gas-phase cooperativity (Δ*G*_ϕ,gas_^*s*^)­

13 $$\Delta G_{\phi }=\Delta \Delta G_{\phi ,\mathrm{geo}}^{s}+\Delta \Delta G_{\phi ,\mathrm{solv}}^{s}+\Delta G_{\phi ,\mathrm{gas}}^{s}$$

with

14 $$\Delta \Delta G_{\phi ,\mathrm{geo}}^{s}=-\Delta G_{\mathrm{geo},\mathrm{ALB}}^{s}+\left(\Delta G_{\mathrm{geo},\mathrm{AL}}^{s}+\Delta G_{\mathrm{geo},\mathrm{BL}}^{s}+\Delta G_{\mathrm{geo},\mathrm{AB}}^{s}\right)-\left(\Delta G_{\mathrm{geo},A}^{s}+\Delta G_{\mathrm{geo},B}^{s}+\Delta G_{\mathrm{geo},L}^{s}\right)$$

15 $$\Delta \Delta G_{\phi ,\mathrm{solv}}^{s}=\Delta G_{\mathrm{solv},\mathrm{ALB}}^{s}-\left(\Delta G_{\mathrm{solv},\mathrm{AL}}^{s}+\Delta G_{\mathrm{solv},\mathrm{BL}}^{s}+\Delta G_{\mathrm{solv},\mathrm{AB}}^{s}\right)+\left(\Delta G_{\mathrm{solv},A}^{s}+\Delta G_{\mathrm{solv},B}^{s}+\Delta G_{\mathrm{solv},L}^{s}\right)$$

16 $$\Delta G_{\phi ,\mathrm{gas}}^{s}=G_{\mathrm{ALB},\mathrm{gas}}^{s}-\left(G_{\mathrm{AL},\mathrm{gas}}^{s}+G_{\mathrm{BL},\mathrm{gas}}^{s}+G_{\mathrm{AB},\mathrm{gas}}^{s}\right)+\left(G_{A,\mathrm{gas}}^{s}+G_{B,\mathrm{gas}}^{s}+G_{L,\mathrm{gas}}^{s}\right)$$

We introduce the *stabilized geometric space* to describe common geometries shared by the individual molecules, binary complexes, and the ternary complex, indicated by superscript “*s*” (middle row of Figure 2A). Δ*G*_geo,*X*_^*s*^ is the geometric free-energy cost bringing molecule *X* from the free space to the stabilized geometric space. Δ*G*_solv,*X*_^*s*^ and Δ*G*_*X*,gas_^*s*^ are the solvation free energy and the gas-phase cooperative free energy of molecule *X* in the stabilized geometric space, respectively. Note that Δ*G*_ϕ_ in eq 13 does not distinguish between *A*, *B*, and *L*, and is invariant under molecular permutation.

**Figure 2:**
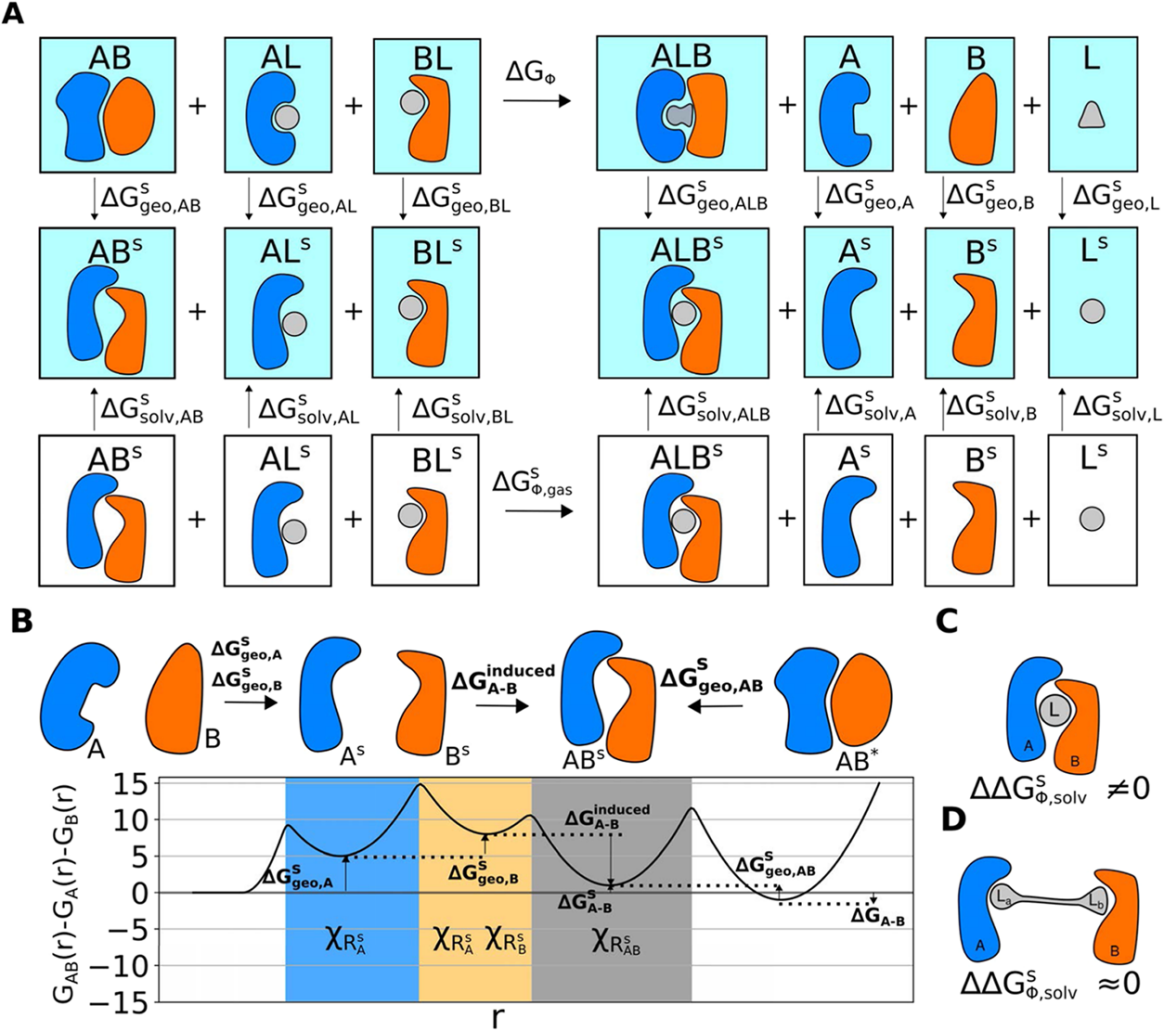
Description of the reduced cooperative free energy ϕ. (A): Thermodynamic cycle of the reduced cooperative free energy. The superscript *s* denotes the *stabilized configurational space*. Δ*G*_geo_^*s*^ is the free-energy change required to bring the free molecule from the global configurational space to the stabilized configurational space and Δ*G*_solv_^*s*^ is the solvation free energy in the stabilized configurational space. (B): Schematic illustration of a free energy profile of the binding process *A* + *B* → *A* ^*s*^ + *B* ^*s*^ → *AB* ^*s*^ → AB*. *A* ^*s*^ and *B* ^*s*^ are the stabilized proteins, *AB* ^*s*^ is the stabilized binary state, and AB* is the global binary state. The blue, yellow, and gray shades represent the stabilized geometric space defined by the indicator functions χ_*R*_*A*_^*s*^_, χ_*R*_*B*_^*s*^_, and χ_*R*_*AB*_^*s*^_, respectively. **r** in the *x*-axis represents the configurational space of the two proteins. (C): Example with nonzero cooperative solvation contribution in the ternary complex with a three-body interface. (D): Example of negligible cooperative solvation contribution ΔΔ*G*_ϕ,solv_^*s*^ in the ternary complex without a three-body interface.

In the following, we will introduce the physical concepts underlying each term in eq 13 by starting with the binary case and extending it to the ternary one. However, due to the asymmetric nature of cooperativity, binary complexation between *A* and *B* is always used as an example in the following context to elucidate the physical meaning of each contribution of Δ*G*_ϕ_.

#### Geometric Free-Energy Costs

In a binary protein–ligand complexation process, a geometric free-energy cost, often called conformational or preorganizational free energy, is the free-energy change required to bring the apo conformation to the holo conformation of the molecules. Similarly, geometric free energy is also associated with a binary complex to change from its energetically minimum state to another configuration to recruit the third body during ternary complex formation. However, this geometric free energy is not captured in binary binding free energy and needs to be described additionally.

When forming a binary complex between two proteins, the binding free energy between the individual proteins is the free-energy difference between the binary complex (*R*_*AB*_) and unbound proteins (*R*_*A*_ and *R*_*B*_). If there exists a predominant bound-state geometry *R*_AB_^*^, for which the partition function dominates the entire bound-state ensemble, the binding free energy can be approximated as the free-energy difference between the unbound state and the dominant bound state (depicted on the two ends of Figure 2B)

17 $$\Delta G_{A-B}=G_{\mathrm{AB}}-G_{A}-G_{B}\approx G_{\mathrm{AB}}^{*}-G_{A}-G_{B}$$

However, in the presence of a stabilizer, the stabilized binary state *R*_*AB*_^*s*^ does not necessarily predominate the entire binary-state ensemble. The stabilized binding free energy is given as

18 $$\Delta G_{A-B}^{s}=G_{\mathrm{AB}}^{s}-G_{A}-G_{B}=\Delta G_{A-B}+\left(G_{\mathrm{sAB}}^{s}-G_{\mathrm{AB}}\right)\geq \Delta G_{A-B}$$

The stabilized geometry of a binary complex *AB* can be defined through an indicator function, χ_*R*_*AB*_^*s*^_(*r*_*AB*_)­

$$\chi_{R_{\mathrm{AB}}^{s}}\left(r_{\mathrm{AB}}\right)=\{\begin{array}{l} 1,\;\;\;\mathrm{if}\;r_{\mathrm{AB}}\;\mathrm{is}\;\mathrm{in}\;\mathrm{the}\;\mathrm{stabilized}\;\mathrm{configurational}\;\mathrm{space}\\ 0,\;\;\;\mathrm{otherwise} \end{array}$$ 19

The free-energy change required to confine the intramolecular geometry of a protein and the intermolecular geometry between multiple proteins from the free space (*G*_*X*_) to the stabilized space (*G*_*X*_^*S*^) is the free-energy difference between the free ensemble and the stabilized ensemble (Figure 2B), i.e.

20 $$\Delta G_{\mathrm{geo},A}^{s}=G_{A}^{s}-G_{A}$$

21 $$\Delta G_{\mathrm{geo},B}^{s}=G_{B}^{s}-G_{B}$$

22 $$\Delta G_{\mathrm{geo},\mathrm{AB}}^{s}=G_{\mathrm{AB}}^{s}-G_{\mathrm{AB}}$$

Since free forms of the proteins are always more favorable than stabilized forms in terms of free energy, the stabilization process introduces a structural and an accompanying thermodynamic perturbation. Hence, we call the processes *unary perturbation* (Δ*G*_geo,*A*_^*s*^ and Δ*G*_geo,*B*_^*s*^) and *binary perturbation* (Δ*G*_geo,*AB*_^*s*^) in the following context.

Rewriting eq 18 with (eqs 20–22) leads to

23 ΔGA−Bs=(GAs−GA)+(GBs−GB)+(GABs−GAs−GBs)=ΔGgeo,As+ΔGgeo,Bs+ΔGA−Binduced

Here, the induced PPIs (Δ*G*_*A*–*B*_^induced^) account for the free-energy difference between stabilized binary complex and stabilized protein

24 $$\Delta G_{A-B}^{\mathrm{induced}}=G_{\mathrm{AB}}^{s}-\left(G_{A}^{s}+G_{B}^{s}\right)$$

It is important to note the difference between the binding free energy (Δ*G*_*A*–*B*_), the binding free energy in the stabilized state (Δ*G*_*A*–*B*_^*s*^), and the induced PPIs (Δ*G*_*A*–*B*_^induced^). Figure 2B shows an example of a weakly interacting protein pair with an energetically unfavorable stabilized binary state but with strong induced PPIs, i.e., Δ*G*_*A*–*B*_^induced^ ≪ Δ*G*_*A*–*B*_ < 0 < Δ*G*_*A*–*B*_^*s*^. In this example, the stabilized binary state *AB* ^*s*^ is difficult to reach from the individual protein states *A* and *B* because of the net positive free-energy difference due to the high unary perturbation Δ*G*_geo,*A*_^*s*^ and Δ*G*_geo,*B*_^*s*^. In contrast, the induced PPIs only consider the binding between the already perturbed (stabilized) proteins, for instance with the help of ligand binding, to form the stabilized binary state.

Substituting eqs 20–23 into eq 14 gives

25 $$\Delta \Delta G_{\phi ,\mathrm{geo}}^{s}=\Delta \Delta G_{\alpha ,\mathrm{geo}}^{s}+\Delta G_{A-B}^{\mathrm{induced}}-\Delta G_{A-B}$$

where

26 $$\Delta \Delta G_{\alpha ,\mathrm{geo}}^{s}=-\Delta G_{\mathrm{geo},\mathrm{ALB}}^{s}-\Delta G_{\mathrm{geo},L}^{s}+\Delta G_{\mathrm{geo},\mathrm{AL}}^{s}+\Delta G_{\mathrm{geo},\mathrm{BL}}^{s}$$

is the net geometric free-energy cost of all ligand-associated states in the asymmetric thermodynamics of cooperative free energy (of the ligand, see eq 11). A more detailed explanation of the definition of stabilized geometries, indicator functions, and geometric free-energy costs is given in Section S1 in the Supporting Information.

#### Cooperative Solvation Contribution

In binary complexation, a desolvation free energy captures a solvation free-energy difference Δ*G*_solv_ during the binding process. For instance, in the process *A* + *B* → *AB*, the binary desolvation free energy is defined as

27 $$\Delta \Delta G_{\mathrm{desolv},A-B}=\Delta G_{\mathrm{solv},\mathrm{AB}}-\left(\Delta G_{\mathrm{solv},A}+\Delta G_{\mathrm{solv},B}\right)$$

Similarly, in the direct ternary complexation *A* + *B* + *L* → *ALB*, the desolvation free energy is

28 $$\Delta \Delta G_{\mathrm{desolv},3}=\Delta G_{\mathrm{solv},\mathrm{ALB}}-\left(\Delta G_{\mathrm{solv},A}+\Delta G_{\mathrm{solv},B}+\Delta G_{\mathrm{solv},L}\right)$$

The cooperative solvation contribution captures the nonadditive part of the ternary desolvation effect

29 ΔΔGϕ,solv=ΔΔGdesolv,3−(ΔΔGdesolv,A−L+ΔΔGdesolv,B−L+ΔΔGdesolv,A−B)=ΔGsolv,ALB−(ΔGsolv,AL+ΔGsolv,BL+ΔGsolv,AB)+(ΔGsolv,A+ΔGsolv,B+ΔGsolv,L)=ΔΔGdesolv,AB−L−(ΔΔGdesolv,A−L+ΔΔGdesolv,B−L)

The last definition of eq 29 indicates that ΔΔ*G*_ϕ,solv_ accounts for the difference between the desolvation free energy of the unary-to-binary binding and the sum of the desolvation free energies from two pairwise binding events. In other words, the cooperative solvation contribution might be nonzero if the two ligand–protein binding interfaces are spatially close to each other (Figure 2C), and becomes gradually negligible when the two interfaces get further apart with the two desolvation events being independent (Figure 2D) of each other. In the rigid body and fixed-charge description, semianalytical methods can further show that ΔΔ*G*_ϕ,solv_ stems predominantly from atoms at the three-body interface (see Section S3 in the Supporting Information). In the stabilized conformational space, the cooperative solvation contribution is described by ΔΔ*G*_ϕ,solv_^*s*^.

#### Gas-Phase Contribution

The gas-phase contribution of the reduced cooperative free energy describes the correlation of individual interactions between one molecule and the other two molecules without a contribution from the solvent (see derivation in Section S4 in the Supporting Information).

### Approximating the Cooperative Free Energy with Induced PPIs and Cooperative Solvation Free Energy

Substituting eq 12 with eqs 13 and 25, the cooperative free energy can be reformulated as,

30 ΔGα=ΔGϕ+ΔGA−B=ΔGA−Binduced+ΔΔGϕ,solvs+ΔGϕ,gass+ΔΔGα,geos≈ΔGA−Binduced+ΔΔGϕ,solvs

Here, a formula that approximates the cooperative free energy is proposed under two assumptions:

1 The two protein–ligand binding events are uncorrelated in the gas phase (Δ*G*_ϕ,gas_^*s*^ ≈ 0).
2 The cooperative geometric contribution is negligible because for a rigid ligand the conformational free-energy cost from the free-form to the ternary form is equal to the sum of the ones from the free-form to the two binary complexes (i.e., ΔΔ*G*_α,geo_^*s*^ = (Δ*G*_geo,*AL*_^*s*^ – Δ*G*_geo,*L*_^*s*^) + (Δ*G*_geo,*BL*_^*s*^ – Δ*G*_geo,*L*_^*s*^) – (Δ*G*_geo,*ALB*_^*s*^ + Δ*G*_geo,*L*_^*s*^) ≈ 0).

The approximated cooperative free energy only considers stabilized geometries and enables an efficient way of estimating cooperativity by evaluating the configurational space near the energy minimum, which is usually well represented in the experimentally resolved ternary structures (see Methods section). To evaluate the strength of induced PPIs and the cooperative solvation contribution in ternary complexes, MD simulations were performed to study 22 ternary complexes, including two PPI stabilizers (PPIS), ten molecular glues, and ten bifunctionals (Table 1). In most of the investigated ternary complexes, the magnitude of the ternary solvation contribution is negligible in comparison to the induced PPIs, except for the complex formed by 14–3–3/ChREBP/compound 3 (PDB ID: 6ygj,^49^ Figure 3A,B), in which the three-body interface involves several atoms from the ligand and the proteins with high partial charges (Figure 3C).

**Table 1:** Protein-Ligand-Protein Ternary Complexes Studied in the Present Study^a^

type	PDB ID	proteins *A*/*B*	ligand *L*	α*	refs
PPIS	3bbr	iGluR2/iGlR2	(*R*,*R*)-2a		^43^
	6mg5	lambda-6A/lambda-6A	coumarin		^44^
molecular glue	5j8o	PD1L/PD1L	BMS-8		^45^
	5j89	PD1L/PD1L	BMS-202		^45^
	5fqd	CRL4/CK1α	lenalidomide		^46^
	3m50	14–3–3/PMA2	epibestatin		^47^
	3m51	14–3–3/PMA2	pyrrolidone1		^47^
	2o98	14–3–3/H-ATPase	fusicoccin-A		^48^
	6ygj	14–3–3β/ChREBP	compound 3	$\alpha_{\mathrm{app}}=\frac{K_{d}^{2}}{K_{d,\mathrm{app}}^{3}}=14$	^49^
	6nv2	14–3–3σ/pS45	DP005	$\alpha_{\mathrm{app}}=\frac{K_{d}^{2}}{K_{d,\mathrm{app}}^{3}}=100$	^39^
	4mdk	CDC34/Ubiquitin-E2	CC0651		^50^
	1kkq	PPARα/MRT corepressor	GW6471		^51^
bifunctional	5ad2	BRD4/BRD4	compound 6		^52^
	5ad3	BRD4/BRD4	compound 2		^52^
	5t35	BRD4^*BD2*^/pVHL	MZ1	α_abs_ = 17.6	^13^
	6sis	BRD4^*BD2*^/pVHL	PROTAC1	α_abs_ = 20	^53^
	6bn7	BRD4^*BD1*^/CRBN	dBET23	$\alpha_{\mathrm{app}}=\frac{{\mathrm{IC}}_{50}^{2}}{{\mathrm{IC}}_{50}^{3}}=0.4$	^25^
	6boy	BRD4^*BD1*^/CRBN	dBET6	$\alpha_{\mathrm{app}}=\frac{{\mathrm{IC}}_{50}^{2}}{{\mathrm{IC}}_{50}^{3}}=0.6$	^25^
	6hay	SMARCA2/pVHL	PROTAC1		^54^
	6hr2	SMARCA2/pVHL	PROTAC2		^54^
	7jto	WDR5/VCB	MS33	α_abs_ = 1.66	^55^
	7jtp	WDR5/VCB	MS67	α_abs_ = 2.74	^55^

a The structures and PDB identifiers are obtained from RCSB PDB.^56^ PPI stabilizers (PPIS) are two cases where the proteins have strong intrinsic PPIs. Molecular glues are compounds that bind to two proteins with weak intrinsic PPIs. Bifunctionals are compounds that possess two protein-binding warheads connected by a covalent linker. [*] Cooperativity values are shown either as absolute cooperativity (α_abs_) where *K*_*d*_^2^/*K*_d_^3^ is measured with isothermal titration calorimetry (ITC), or as apparent cooperativity (α_app_) calculated by *K*_*d*_^2^/*K*_d,app_^3^, where *K*_d,app_^3^ is the apparent dissociation constant, or IC_50_^2^/IC_50_^3^.

**Figure 3:**
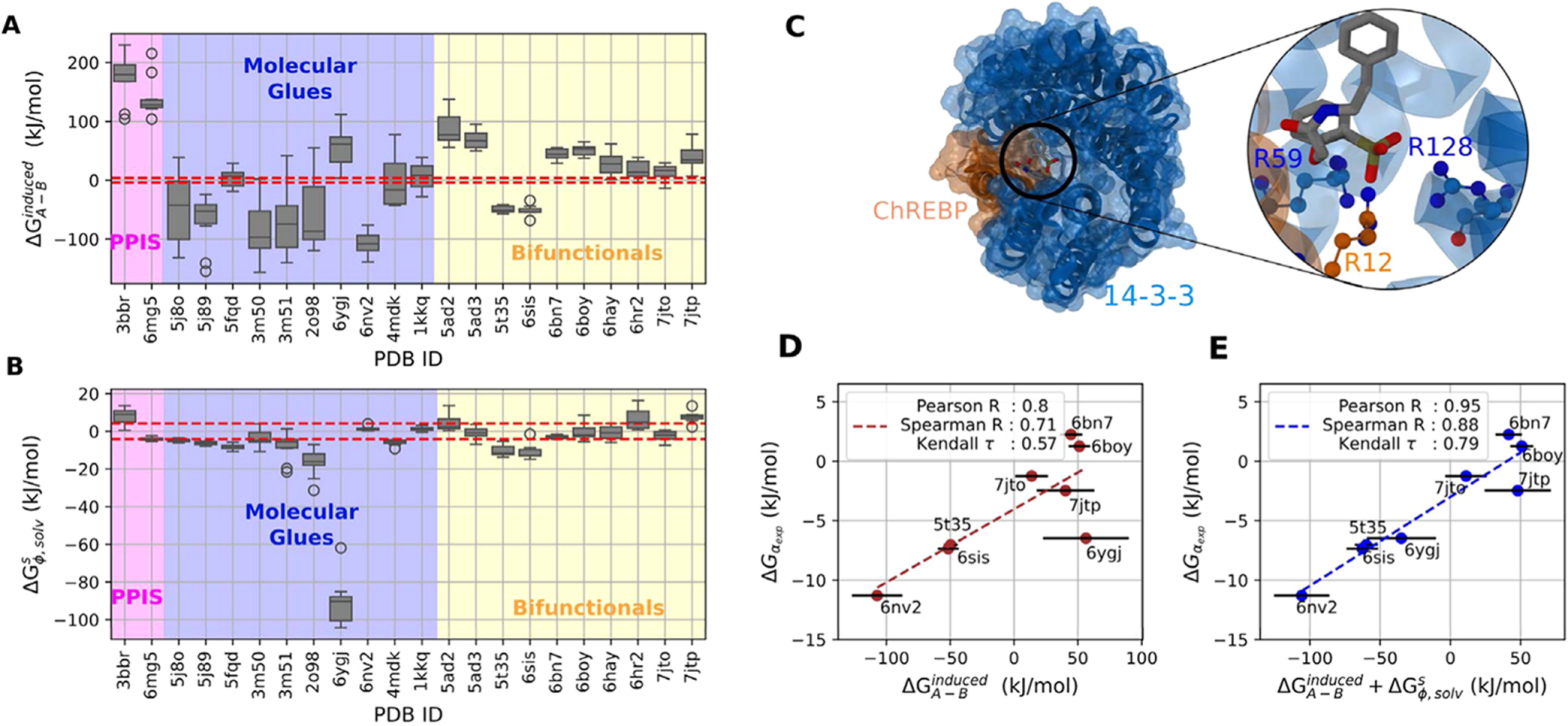
Approximated cooperative free energy estimation from MD simulations. (A): Calculated induced PPI contribution for the 22 ternary complexes listed in Table 1. (B): Cooperative solvation contribution for these complexes. (C): The highly charged three-body interface between protein 14–3–3 (blue), protein ChReBP (orange), and ligand compound 3 (gray) (PDB ID: 6ygj). The zoom-in view highlights the charged residues at the interface, of which the carbon atoms are colored with respect to their residue. (D): Comparison between experimental apparent cooperativity and approximated cooperative free energy based on calculated induced PPIs. The dashed line shows the regression fit *y* = −0.96 + 0.06*x*. (E): Same comparison with the approximated cooperative free energy based on the sum of calculated induced PPIs and cooperative solvation free energy using MM/PBSA. The dashed line shows the regression fit *y* = – 0.72 + 0.07*x*. The red dashed lines in (A, B) show the ± 4.18 kJ/mol (±1 kcal/mol) interval. The error bars in subplots (A, B, D, E) show the standard deviation of the mean (*N* = 10).

Next, we compared the calculated approximated cooperativity with the experimental cooperativity values for the eight ternary complexes for which such data were available (Table 1 and Figure 4). In most cases, induced PPIs alone are enough to show a correlation with apparent cooperativity (Figure 3D). We note that the underestimated cooperativity of complex WDR5/VCB/MS67 (PDB ID: 7jtp)^55^ can be attributed to the abundant water-mediated hydrogen bonds at the protein–protein interface (Figure S5 in the Supporting Information), which is not captured in the solvation free-energy estimation using the implicit solvation method. Nonetheless, including ternary solvation free energy successfully corrects the calculated cooperativity value of complex 14–3–3/ChREBP/compound 3 (PDB ID: 6ygj)^49^ and increases the overall agreement of cooperativity prediction (Figure 3E).

**Figure 4:**
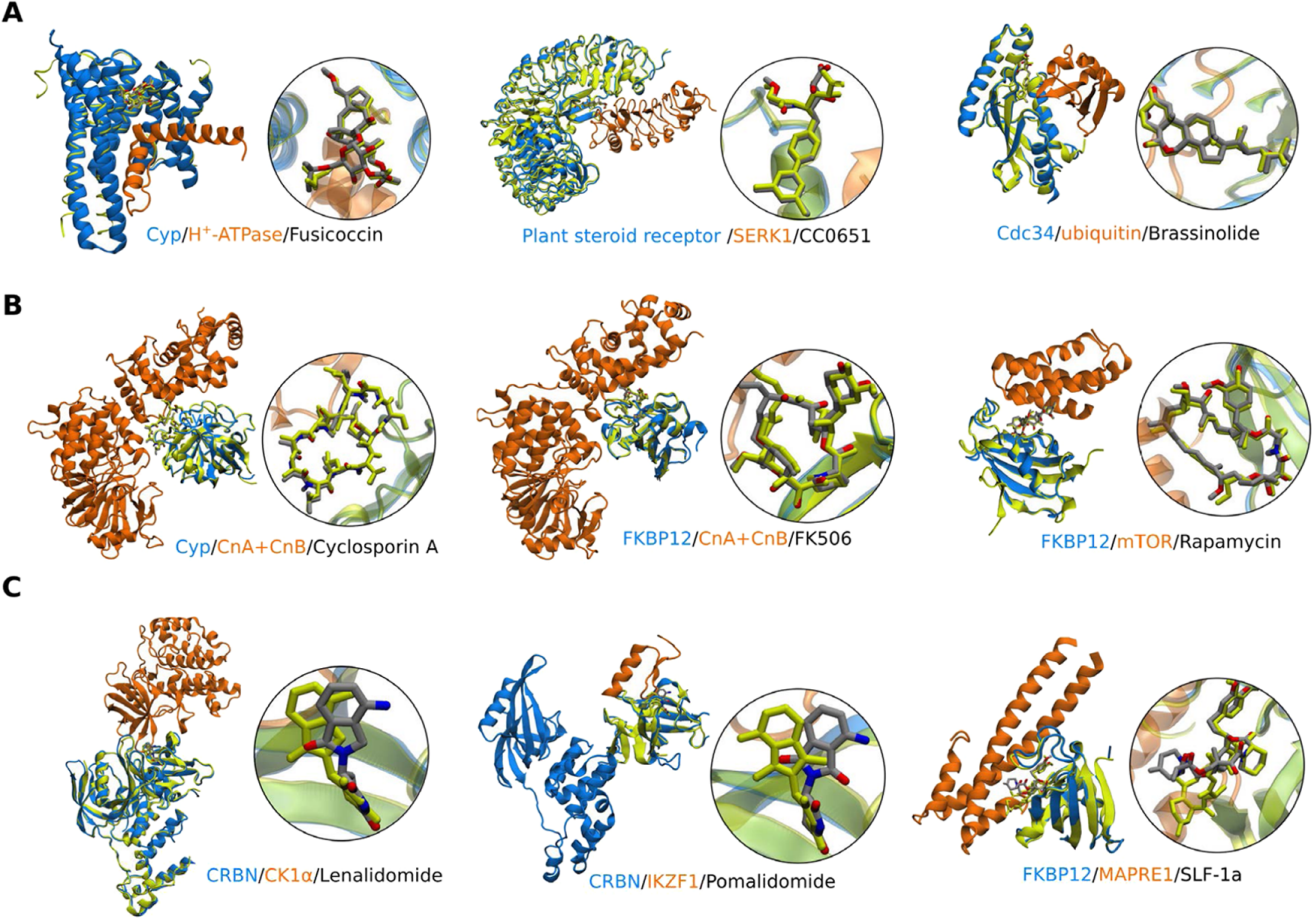
Superposition of crystal structures of the protein–ligand binary complex (yellow) and the protein–ligand–protein ternary complex (blue-gray-orange). (A): Examples of noncyclic small molecules with the same binding pose. Structures from left to right (binary/ternary): 5mxo^57^/2o98,^48^ 3rz3 ^31^/4mdk,^50^ and 4lsa^58^/4lsz.^59^ (B): Examples of macrocycles with the same binding pose. Structures from left to right: 1cwc^60^/1mf8,^61^ 5hw8 ^62^/6tz6,^63^ and 4qt3 ^64^/1fap.^65^ (C): Examples with different binding poses. Structures from left to right: 4tzt^66^/5fqd,^46^ 4v2z^67^/6h0f,^68^ and 9co5 ^69^/9dcw.^69^

Despite the good correlation between the approximated cooperative free energy and experiment for the eight examples, we would like to stress that the assumption of fixed protein–ligand conformation may sometimes be violated. While many protein–ligand complexes have similar ligand binding poses in binary and ternary forms with small molecules (e.g., fusicoccin,^48,57^ CC0651,^31,50^ and brassinolide,^58,59^ see Figure 4A) or macrocycles (e.g., cyclosporin A,^60,61^ FK506,^62,63^ and rapamycin,^64,65^ see Figure 4B), other ligands show a different binding mode between binary and ternary complex or involve a conformational transition of the protein to recruit the third binding partner (e.g., lenalidomide,^46,66^ pomalidomide,^67,68^ and SLF-1a,^69^ see Figure 4C). Note that the structure of the binary complex pomalidomide-CRBN shown in Figure 4 is chain C in PDB 4v2z to show a potentially dramatic difference between the binary and ternary ligand binding mode, while chain A and B of 4v2z demonstrate a binding pose similar to the one in the lenalidomide-CRBN complex.^67^ Therefore, for flexible ligands the approximation of a zero cooperative geometric contribution may have to be revisited when using the computational protocol present in this study.

### Relationship between Induced PPIs and Cooperativity

Our computational study suggests that cooperativity—in most cases—is dominated by induced PPIs. Therefore, it is important to understand the composition of induced PPIs and how to modulate them. Rewriting eq 23 with eqs 18 and 17 provides a better understanding of how the induced PPIs are modulated by the ligand

31 $$\Delta G_{A-B}^{\mathrm{induced}}-\Delta G_{A-B}=\Delta G_{\mathrm{geo},\mathrm{AB}}^{s}-\left(\Delta G_{\mathrm{geo},A}^{s}+\Delta G_{\mathrm{geo},B}^{s}\right)$$

The corresponding energy-geometric process is depicted in Figure 2B. In eq 31, Δ*G*_*A*–*B*_ corresponds to the intrinsic PPIs unique to any protein pair that bring the two unbound proteins to the energetically favorite binary state, i.e., the binary state with the lowest free energy, even if Δ*G*_*A*–*B*_ is positive (Figure 5A). The strength of induced PPIs depends on the interplay between unary (Δ*G*_geo,*A*_^*s*^ + Δ*G*_geo,*B*_^*s*^) and binary perturbation (Δ*G*_geo,*AB*_^*s*^). An example of a unary perturbation is the change in the secondary structure upon binding of a molecular glue (Figure 5B), whereas an example of a binary perturbation is the disruption of native protein–protein contacts by PPI stabilizers (Figure 5C).

**Figure 5:**
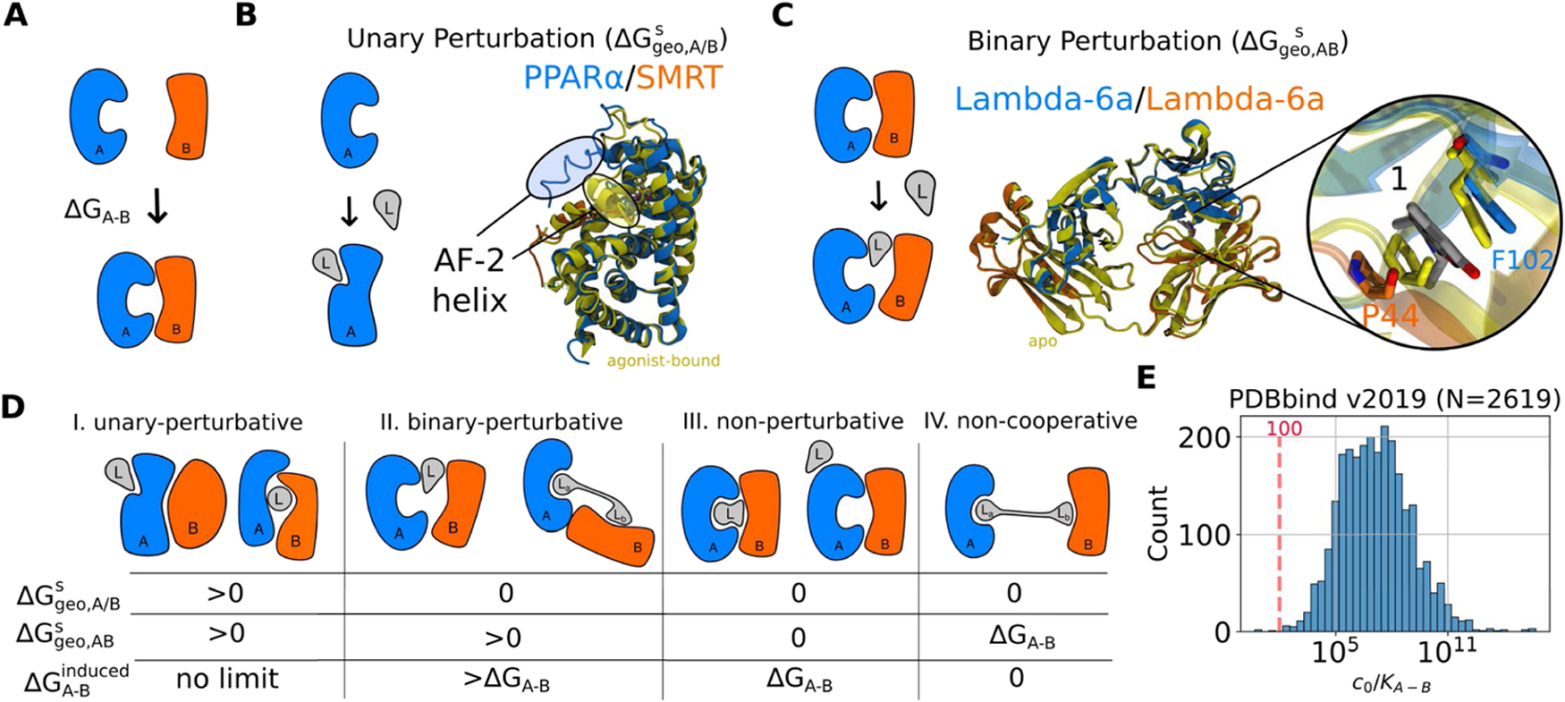
Geometric perturbation and induced PPIs in ternary complexes. (A): Example of a binding event between two proteins *A* and *B*. (B): An example of a unary perturbation is the rigidification of the secondary structure of the AF-2 helix of PPARα (blue) upon binding of the molecular glue GW6471 (gray, PDB ID: 1kkq^51^) to recruit SMRT (orange). The agonist-bound structure is overlaid (yellow, PDB ID: 1k7l^70^). (C): An example of a binary perturbation is the disruption of the intrinsic PPIs of the Lambda-6a dimer (blue and orange) by compound 1 (gray, PDB ID: 6mg4 ^44^), compared to the ligand-free dimer (yellow, PDB ID: 6mg5 ^44^). (D): Schematic illustration of four different types of induced PPIs modulated by unary (Δ*G*_geo,*A*/*B*_^*s*^) and binary (Δ*G*_geo,*AB*_^*s*^) perturbations. The induced PPIs (Δ*G*_*A*–*B*_^induced^) are computed according to eq 31. (E): Statistics of the PPIs from 2619 1/*K*_d_ values of structurally resolved protein–protein complexes from PDBbind v2019.^71,72^ *c*_0_ is the standard concentration of 1 molar and *K*_A–B_ is used here to distinguish the protein–protein affinities from ligand-protein affinities.

Briefly, Figure 5D depicts how the induced PPIs are modulated by four types of geometric perturbations from the unique bound state shown in Figure 5A. A ligand that causes a unary perturbation can lead to induced PPIs stronger or weaker than the intrinsic PPIs, depending on the respective strength of the unary and binary perturbation in eq 31. A “binary-perturbative” ligand does not influence the intramolecular geometry of individual proteins but alters the intermolecular geometry between two proteins to another binding pose. This induces new PPIs that must be weaker than the intrinsic PPIs. With a nonperturbative ligand, which binds at any surface of the two proteins without perturbing the energetically favorite binary state, induced PPIs are equal to the intrinsic PPIs. Lastly, a noncooperative ligand that separates the two proteins back to the binary state essentially reverses the binding process and leads to zero induced PPIs. Thus, nonperturbative and noncooperative modulation of cooperativity become optimal solutions for proteins with negative and positive intrinsic PPIs without unary perturbation, respectively.

Next, we aim to understand how strong induced PPIs need to be to introduce high cooperativity. In systems where only induced PPIs contribute to the cooperative free energy, one can approximate cooperativity with the effective protein–protein dissociation constant from eq 11

32 α=exp(−βΔGα)≈exp(−βΔGA−Binduced)=[ABs]c0[As][Bs]=c0/KA−Binduced

eq 32 demonstrates an inconsistency between what is thought to be a “weak” protein–protein binding affinity (*K*_*A*–*B*_ > 1 μM^73,74^ ) and a “strong” cooperativity (α > 10^2^). Indeed, 99.89% of the 2619 structurally resolved protein–protein pairs recorded in the PDBbind database^71,72^ possess a *K*_*A*–*B*_ stronger than 10 mM (Figure 5E). However, without inducing any *de novo* PPIs, a nonperturbative ligand obtains a cooperativity of 100 when binding at any surface of a weakly interacting protein pair, even if it does not stabilize the ternary complex at all (see eq 32 and Figure 5D). One quickly notices that this “cooperative” ligand does not in fact introduce any cooperative phenomenon but merely reveals the intrinsic PPIs. This counter-intuitive finding comes from the asymmetric nature of cooperativity (of the ligand) and becomes clear when the effective ternary dissociation constant in eq 1 is written with reduced cooperativity ϕ instead of cooperativity α

33 K3=α−1KA−LKB−L=ϕ−1KA−BKA−LKB−L

Here, ϕ is 1 for a nonperturbative ligand and ϕ = *K*_*A*–*B*_ for the cooperative phenomenon introduced by noncooperative PROTACs (Figure 5D). In fact, binding a ligand between two proteins does not only modulate the stabilized geometry by unary and binary perturbations but also via solvation perturbation. However, including the solvation perturbation in a pairwise manner requires symmetry to describe the cooperative process. In Section S5 in the Supporting Information, we derive a symmetric representation of physical cooperativity and show how solvation perturbation influences the effective binding free energy between two proteins (Figure S4 in the Supporting Information). For simplicity, we do not extend the discussion on solvation perturbation and focus only on induced PPIs.

### Comparison between Ligand-Induced Protein–Protein Binding Poses

Despite the unresolvable intrinsic binding poses of weakly interacting proteins, it is possible to compare how the same protein pairs are stabilized in well-aligned poses and when bound by different compounds assuming no ternary perturbation, i.e., Δ*G*_geo,*ALB*_^*s*^ = 0 (Figure 2A). Figure 6 shows four examples of well-aligned protein–protein binding poses and four examples of the latter. Surprisingly, despite the distinctly different scaffolds of epibestatin and pyrrolidone1 (Tanimoto similarity = 0.11 with Morgan fingerprint^75^ with a radius of 2) and different binding locations (ligand center-of-mass (COM) deviation = 0.99 nm), they show a very similar intermolecular geometry between proteins 14–3–3 and PMA2 (Figure 6A). Similarly, compounds BMS-8 and BMS-22 (Tanimoto similarity = 0.33) bind at different interfaces of the PDL1 homodimer (ligand COM deviation = 1.24 nm), yielding similar dimer configurations (Figure 6A). The same protein–protein binding poses are also observed when stabilized by compounds that bind at the same site such as (R)-CR8 and HQ461 (Tanimoto similarity = 0.13) binding to DDB1/CDK12 (ligand COM deviation = 0.15 nm) or two PROTACs with the same warheads and similar linker length binding to pVHL/SMARCA2 (Tanimoto similarity = 0.86) (Figure 6A).

**Figure 6:**
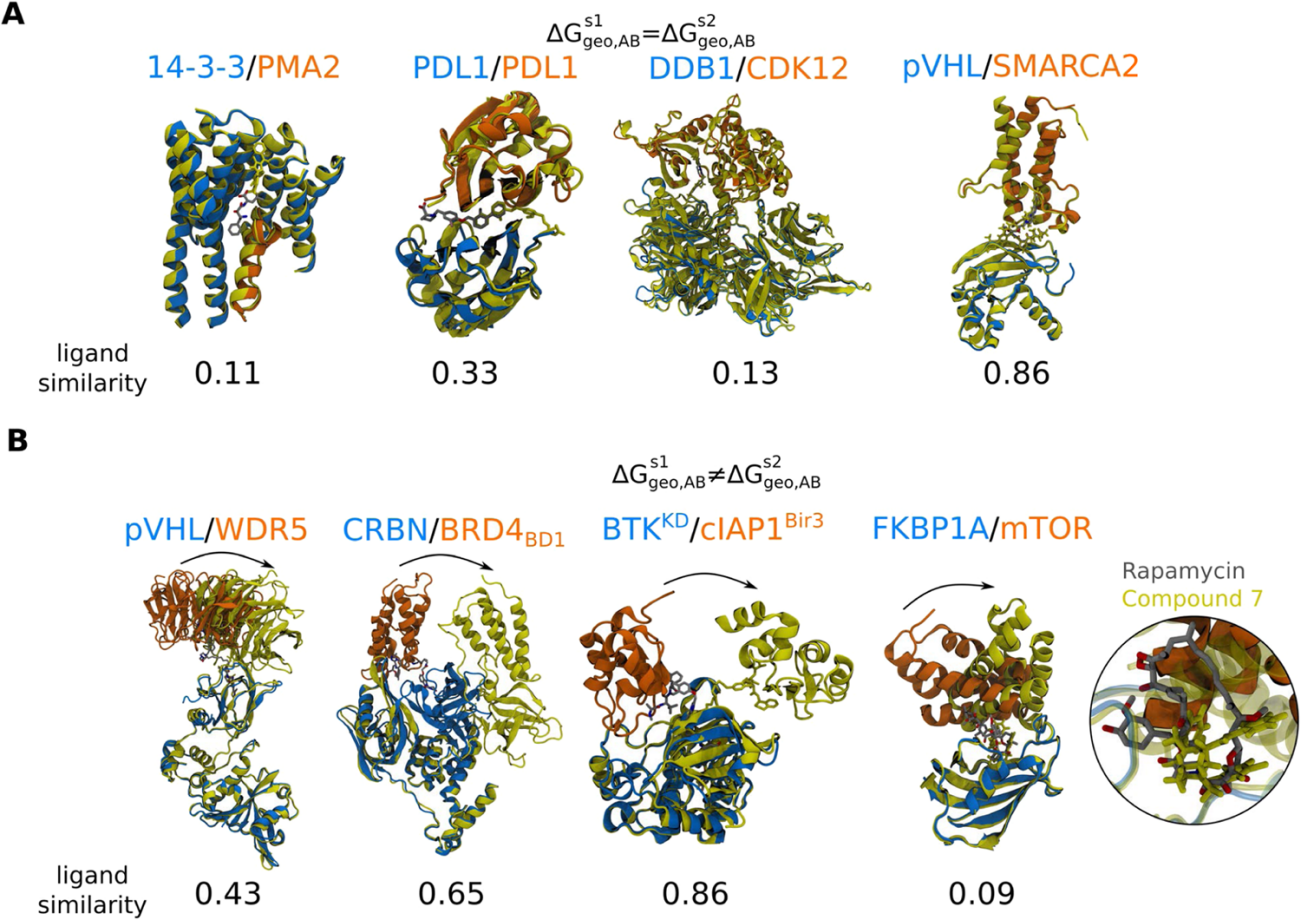
Superposition of crystal structures of ternary complexes of the same protein pairs with different ligands. Proteins of the first complex are shown in blue and orange, and the ligand in gray. The second complex is shown all in yellow. Δ*G*_geo,*AB*_^*s*1^ and Δ*G*_geo,*AB*_^*s*2^ are the geometric free energies to perturb the intrinsic PPIs of the first and second stabilized binary complexes, respectively. (A): Examples with similar protein–protein binding poses (from left to right): 14–3–3/PMA2 with epibestatin (PDB ID: 3m50 ^47^) and pyrrolidone1 (PDB ID: 3m51 ^47^), PDL1 dimer with BMS-8 (5j8o^45^) and BMS-202 (5j89 ^45^), DDB1/CDK12 with (R)-CR8 (6td3 ^76^) and HQ461 (8bug^77^), pVHL/SMARCA2 with PROTAC 1 (6hay^54^) and PROTAC 2 (6hr2 ^54^). (B): Examples with differently stabilized protein–protein binding poses of the same protein pair (from left to right): pVHL/WDR5 with MS33 (7jto^55^) and MS67 (7jtp^55^), CRBN/BRD4_DB1_ with dBET23 (6bn7 ^25^) and dBET57 (ligand is not resolved in the experiment, 6bnb^25^), BTK^KD^/cIAP1^Bir3^ with BCPyr (6w7o^78^) and BC5P (6w8i^78^), and FKBP1A/mTOR with rapamycin (1fap^65^) and compound 7 (8ppz^79^). Ligand similarity was calculated with Morgan bit fingerprints^75^ with a radius of 2 and Tanimoto similarity as implemented in the RDKit.^80^ Chemical structures of the compounds are shown in Figure S4 in the Supporting Information.

In contrast, when bound by different compounds, different protein–protein binding poses of the same protein pair are observed upon binding of PROTACs with the same warheads, but different linker scaffolds (Figure 6B). Intriguingly, despite the high similarity between compounds dBET23 and dBET57 (Tanimoto similarity = 0.90), they induce both binary and unary perturbations of the CRBN conformation. Besides PROTACs, the binding poses between two proteins such as FKBP1A and mTOR can also deviate upon the binding of macrocyclic molecular glue rapamycin or the small-molecule molecular glue compound 7 (Figure 6B).

## Conclusions

In ternary complex formation, cooperativity (of the ligand) is an asymmetric quantity describing how binding of a ligand to the first protein facilitates binding of a second protein. In addition to the binary ligand–protein binding affinities, cooperativity stands out as the third factor determining the efficiency of ternary complexation by the three partners. While high ligand–protein binding affinities can lead to an unwanted hook effect, cooperativity monotonically increases ternary complex formation. Increasing cooperativity at the cost of binary binding affinity can lead to an equally high fraction of ternary complex formation, while alleviating the hook effect. To facilitate a physical interpretation of cooperativity, we introduced the cooperative free energy, which is the sum of the protein–protein binding free energy and all nonpairwise additive effects during a three-body complex-forming process.

Cooperative free energy can be divided into four contributions by introducing the concept of “stabilized geometric space” in which all unary, binary, and ternary species show a common conformation. First, induced PPIs characterize the protein–protein contacts in the presence of the stabilizer. Second, cooperative solvation free energy accounts for the double-counted desolvation effect from the sum of the two protein–ligand desolvation events at the three-body interface. Third, gas-phase reduced cooperativity originates from the cooperative entropic effect and can be omitted when two ligand–protein binding events are independent in the stabilized geometric space. Lastly, ligand-cooperative conformational free energy accounts for the free-energy cost to bring a ligand from different complexation states to a common stabilized geometric space.

We demonstrate how the approximated cooperative free energy can be estimated from short simulations (10 ns with ten replicates) and correlates with experimental data. By analyzing the thermodynamics of the induced PPIs, we conclude that unless a ligand can induce an unary perturbation to better bind its protein partner, i.e., Δ*G*_geo,*A*_^*s*^ + Δ*G*_geo,*B*_^*s*^ > Δ*G*_geo,*AB*_^*s*^, inducing any *de novo* PPIs only worsens the induced PPIs. For example, preserving the intrinsic weak PPIs with a weak *K*_d_ of 10 mM by a nonperturbative ligand already leads to a cooperativity of 100. It is noteworthy that in the context of protein–protein stabilization the term cooperativity does not directly represent the cooperative phenomenon (i.e., nonpairwise-additive contributions) as it is commonly understood in mathematics and physics due to its asymmetricity. For instance, a nonperturbative ligand can induce high cooperativity without bringing any three-body effect, whereas a noncooperative ligand is actually cooperative because it strongly modulates how two proteins interact with each other.

Lastly, we discuss the similar and dissimilar protein–protein binding poses of eight pairs of ternary complexes available in the literature and suggest that nonperturbative ligands might already stabilize the intrinsic PPIs. We anticipate that our work and the new insights into understanding the cooperative process in ternary complexes will facilitate future design of cooperative PPI stabilizers.

## Methods

### Structure Preparation and MD Simulations

Only the coordinates of the two proteins and the stabilizer at the ternary interface were considered in the eight PROTACs-mediated ternary complexes (PDB ID: 5t35, 6sis, 6boy, 2hay, 2hr2, 7jto, and 7jtp), i.e., water molecules, ions, and other cocrystallized compounds were ignored. Coordinates of missing and mutated residues from the experimental structures were generated with MODELER v10.4.^81^ The coordinates of the other 14 ternary complexes listed in Table 1 were taken from ref 21.

Each ternary complex was solvated in an OPC water^82^ box with a length 1.25 nm from the protein edge in each dimension with 0.15 M NaCl using the program *tleap* from AmberTools22.^83^ In total, 22 systems were prepared and subjected to energy minimization, equilibration, and production phases with the Amber22 simulation package.^83^ Energy minimization of 70,000 steps was performed with position restraints with a force constant of 0.418 kJ/mol/nm^2^ on the two proteins and the ligand (all atoms). Each minimized structure was equilibrated ten times with different randomly generated initial velocities to generate ten equilibrated structures. Each equilibration consisted of 75 ps NVT and 125 ps NPT simulations with gradually decreasing positional restraints (from 0.418 to 0.004 kJ/mol/nm^2^) on proteins and ligands. An additional 1 ns NPT simulation was performed with a positional restraint of 0.004 kJ/mol/nm^2^ on only the ligand to relax protein side chains. Each equilibrated system was submitted to a 10 ns production run. The temperature was kept at 298 K using a Langevin thermostat,^84^ and the pressure was kept at 1 bar with a Berendsen barostat.^85^ The nonbonded cutoff distance was set to 0.9 nm, and the simulation was integrated with a time step of 2 fs with the SHAKE algorithm^86^ to constrain the bond lengths of bonds involving hydrogen with a relative geometrical tolerance of 10^–6^. The translational and rotational center-of-mass motion was removed every 1000 steps. Trajectories were printed every 5 ps in the production simulation for analysis.

Atomic interactions were described by the force field AMBER ff19SB^87^ for proteins, GAFF2 ^88^ for ligands, OPC^82^ for water molecules, and Joung/Cheatham ion parameters^89^ for ions. Partial charges of the ligands were assigned with the AM1-BCC^90^ method implemented in the program *antechamber*.^91^

### Energy Evaluation

Single-trajectory approximation was applied to all energy analyses within the stabilized conformational space from the ternary complex simulations, namely, for an arbitrary quantity *O*

34 $$\langle O\rangle_{\mathrm{ALB}}\approx \langle O\rangle_{AB^{s}}\approx \langle O\rangle_{AL^{s}}\approx \langle O\rangle_{BL^{s}}\approx \langle O\rangle_{A^{s}}\approx \langle O\rangle_{B^{s}}\approx \langle O\rangle_{L^{s}}$$

The interaction and solvation free energies between the ligand and the proteins were computed with MMPBSA.py.^92^ The solvation energies of the individual molecules, binary complexes, and ternary complexes in Figure 5D were calculated with the molecular mechanics Poisson–Boltzmann surface area (MM/PBSA) method using this python program. As the induced PPIs do not include the conformational change of the free protein, it is possible to directly evaluate its strength by calculating the interaction between the two proteins (*E*_*A*–*B*_) using a single-trajectory approximation, neglecting the binary entropy contribution

35 $$\Delta G_{A-B}^{\mathrm{induced}}=\Delta G_{AB^{s}}-\Delta G_{B^{s}}-\Delta G_{A^{s}}=\langle E_{A-B}\rangle_{\mathrm{AL}B^{s}}+\langle \Delta G_{\mathrm{desolv},\mathrm{AB}}\rangle_{\mathrm{AL}B^{s}}$$

36 $$\langle E_{A-B}\rangle_{\mathrm{AL}B^{s}}=\langle E_{\mathrm{AB}}\rangle_{\mathrm{AL}B^{s}}-\langle E_{A}\rangle_{\mathrm{AL}B^{s}}-\langle E_{B}\rangle_{\mathrm{AL}B^{s}}$$

37 $$\langle \Delta G_{\mathrm{desolv},\mathrm{AB}}\rangle_{\mathrm{AL}B^{s}}=\langle \Delta G_{\mathrm{GB},\mathrm{AB}}-\Delta G_{\mathrm{GB},A}-\Delta G_{\mathrm{GB},B}\rangle_{\mathrm{AL}B^{s}}$$

where Δ*G*_*GB*,*AB*_, Δ*G*_*GB*,*A*_, and Δ*G*_*GB*,*B*_ are the solvation energy of the binary complex *AB* as well as proteins *A* and *B* evaluated using MM/PBSA with the ensemble obtained in the ternary simulation, respectively. The cooperative solvation free energy was calculated via

$$\langle \Delta G_{\mathrm{desolv},\mathrm{AB}}\rangle_{\mathrm{AL}B^{s}}={\left\langle \Delta G_{\mathrm{GB},\mathrm{ALB}}-\Delta G_{\mathrm{GB},\mathrm{AL}}-\Delta G_{\mathrm{GB},\mathrm{BL}}-\Delta G_{\mathrm{GB},\mathrm{AB}}+\Delta G_{\mathrm{GB},A}+\Delta G_{\mathrm{GB},B}+\Delta G_{\mathrm{GB},L}\right\rangle }_{{\mathrm{ALB}}^{s}}$$ 38

MM/PBSA and MM generalized-Born surface area (MM/GBSA) including protein–protein interface water molecules (Nwat-MM/PBSA^93^ and Nwat-MMGB/SA^94,95^ ) were used to estimate experimental cooperativity. However, this did not lead to improved results (Figure S7 in the Supporting Information). The partial charges of the OPC water model were changed to the ones of TIP3P^96^ in the MM/PB­(GB)­SA-Nwat calculations to enable the use of MMPBSA.py.

## Supplementary Material



## Data Availability

PDB files of the ternary complexes, the GAFF2-parametrized ligand parameter files, simulation scripts, and analysis scripts are freely available on the GitHub repository: https://github.com/rinikerlab/cooperative-free-energy.

## Notes

Mathematical derivations and additional figures S1–S7 discussed in the text; response of the ternary-complex concentration [*ALB*] as a function of [*L*_tot_]; thermodynamic cycle of the solute geometric change; difference between the induced PPIs and the effective PPIs in the ternary complexes; other endpoint evaluation methods by including *N* interface water molecules in (A) MM/PBSA and (B) MM/GBSA (PDF)
