# Supplementary material for: Cooperative Free Energy: Induced Protein–Protein Interactions and Cooperative Solvation in Ternary Complexes
Source: J Chem Theory Comput. 2025 Aug 20;21(17):8557–70. doi: 10.1021/acs.jctc.5c00736 (PMC12424184; doi:10.1021/acs.jctc.5c00736)
Supplement: Supplementary file 1 [file ct5c00736_si_001.pdf]

SUPPORTING INFORMATION
Cooperative Free Energy: Induced Protein–Protein Interactions and Cooperative Solvation in Ternary Complexes

Shu-Yu Chen,[a] Riccardo Solazzo,[a] Marianne Fouché,[b] Hans-Jörg Roth,[b] Birger Dittrich,[b] and Sereina Riniker*[a]

[a] *Department of Chemistry and Applied Biosciences, ETH Zürich, Vladimir-Prelog-Weg 2, 8093 Zürich, Switzerland. E-mail: sriniker@ethz.ch*

[b] *Novartis Biomedical Research, Novartis Campus, 4002 Basel, Switzerland*

Contents

S1 Concentration of Ternary Complex at Equilibrium **S2**

S2 Geometric Free-Energy Difference **S3**

S3 Semi-analytical Model for the Estimation of the Cooperative Solvation Contribution **S6**

S4 Estimation of the Gas-phase Contribution to the Reduced Cooperativity **S8**

S5 Symmetric Description of Cooperativity **S9**

S1 Concentration of Ternary Complex at Equilibrium

Considering a system consisting of two weakly interacting biomolecules A and B, adding a ligand L results in binary complexes AL and BL, and ternary complex ALB. The concentration of each species can be expressed in terms of dissociation constants,

$$K_{A-L} = \frac{[A][L]}{[AL]} \tag{1}$$

$$K_{B-L} = \frac{[B][L]}{[BL]} \tag{2}$$

$$K_{AL-B} = \frac{[AL][B]}{[ALB]} = \frac{1}{\alpha} K_{B-L} \tag{3}$$

$$K_{A-BL} = \frac{[A][BL]}{[ALB]} = \frac{1}{\alpha} K_{A-L}, \tag{4}$$

where $[X]$ is the concentration of the species X, and K_{X-Y} is the dissociation constant between species X and Y. In addition, the mass conservation law constrains the concentrations of the individual species,

$$[A_{tot}] = [A] + [AL] + [ALB] \tag{5}$$

$$[B_{tot}] = [B] + [BL] + [ALB] \tag{6}$$

$$[L_{tot}] = [L] + [AL] + [BL] + [ALB]. \tag{7}$$

Here, the concentration of the binary protein complex $[AB]$ is ignored due to the weak interactions between the protein partners. Solving Eqs. 1-7 gives,

$$[L_{tot}] = [L] + \frac{[L][A_{tot}]}{[L] + K_{A-L}} + \frac{[L][B_{tot}]}{[L] + K_{B-L}} + (1 - \frac{[L]}{[L] + K_{A-L}} + \frac{[L]}{[L] + K_{B-L}})[ALB] \tag{8}$$

and

$$[ALB] = \frac{C - \sqrt{C^2 - 4[A_{tot}][B_{tot}]}}{2} \tag{9}$$

$$C \equiv [A_{tot}] + [B_{tot}] + \frac{([L] + K_{A-L})([L] + K_{B-L})}{\alpha[L]}. \tag{10}$$

Differentiating the ternary concentration $[ALB]$ with respect to $[L_{tot}]$ gives,

$$\frac{d[ALB]}{d[L_{tot}]} = (\frac{[ALB]\alpha^{-1}}{2[ALB] - C})(1 - \frac{K_{A-L}K_{B-L}}{[L]^2})\frac{[L]}{[L_{tot}]}, \tag{11}$$

which vanishes at

$$[L] = [L]^{opt} = \sqrt{K_{A-L}K_{B-L}}. \tag{12}$$

Substituting Eq. 12 in Eq. 8, one obtains the optimal ligand concentration

$$[L_{tot}]^{opt} = \sqrt{K_{A-L}K_{B-L}} + \frac{[A_{tot}]}{1 + \sqrt{K_{A-L}/K_{B-L}}} + \frac{[B_{tot}]}{1 + \sqrt{K_{B-L}/K_{A-L}}}. \tag{13}$$

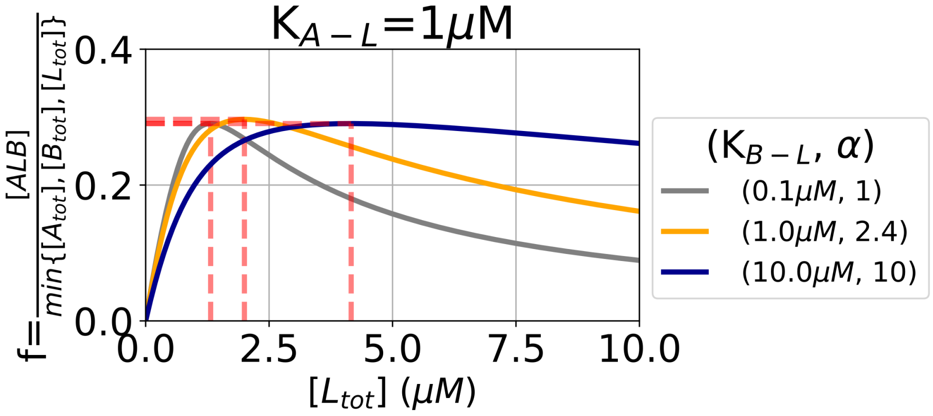

Figure S1: Response of the ternary-complex concentration $[ALB]$ as a function of $[L_{tot}]$. Red dash lines: Demonstration that the same amount of f^{opt} can be reached by a different combinations of K_{B-L} and α with a fixed K_{A-L}.

S2 Geometric Free-Energy Difference

We are aware of the diverse definitions of the terms "conformation", "configuration", "induced fit", and "allostery" and decided to include all of these in the term "geometry". Any geometric change from the free-energy minimum is called "perturbation". The term "configuration" is used here to match the thermodynamics textbooks and refers strictly to a unique arrangement of all atoms in the system.

Considering a system consisting of two solutes A and B surrounded by water molecules, the configuration of the system can be described by the intermolecular geometries of the individual proteins r_A and r_B, the intermolecular geometry between them r_{A-B}, and the distribution of the water molecules r_{wat}. At equilibrium, the probability of the solutes remaining in a configuration r_{sol} follows the Boltzmann distribution

$$P(r_{sol}) = \frac{\int e^{-\beta U(r_{sol},r_{wat})} dr_{wat}}{Z}, \tag{14}$$

where U is the potential energy describing the interaction between the molecules, and Z is the partition function that normalizes the probability over all solute configurations $R = \{r_{sol}\}$. The associated Gibb's free energy of the system is,

$$G_R = -\beta^{-1} \ln Z = -\beta^{-1} \ln \int \int e^{-\beta U(r_{sol},r_{wat})} dr_{wat} dr_{sol}. \tag{15}$$

By introducing an indicator function $\chi^*_R(r_{sol})$ to determine whether a solute configuration r_{sol} belongs to a target geometric space R^*, the free energy of the subspace is,

$$G_{r_{sol} \in R^*} = -\beta^{-1} \ln \int \int e^{-\beta U(r_{sol},r_{wat})} \chi^*_R(r_{sol}) dr_{wat} dr_{sol} \tag{16}$$

$$\chi_{R^*}(r_{sol}) = \begin{cases} 1, & \text{if } (r_A, r_B, r_{A-B}) \in R^*_A \times R^*_B \times R^*_{A-B} \\ 0, & \text{otherwise,} \end{cases} \tag{17}$$

where R_A^*, R_B^*, and R_{A-B}^* indicate intramolecular and intermolecular geometries in the target space. Equivalently, Eq. 17 can be expressed as,

$$\chi_{R^*}(r_{sol}) = \chi_{R_A^*}(r_A) \times \chi_{R_B^*}(r_B) \times \chi_{R_{A-B}^*}(r_{A-B}). \tag{18}$$

The free energy of the conformations out of the subspace is,

$$G_{r_{sol} \notin R^*} = -\beta^{-1} \ln \int \int e^{-\beta U(r_{sol}, r_{wat})}[1 - \chi_{R^*}(r_{sol})] dr_{wat} dr_{sol}. \tag{19}$$

An example of such target space is the bound state of proteins A and B, where the indicator function defines the bound state R_{AB}. In this case, the indicator function can be determined by an experimental setup such as the minimal Förster resonance energy transfer (FRET) distance,

$$\chi_{FRET,R_{AB}}(r_{sol}) = \begin{cases} 1, & \text{if } d_{FRET} < d_{FRET}^{min} \\ 0, & \text{otherwise.} \end{cases} \tag{20}$$

Here, d_{FRET} is the distance between the FRET donor and acceptor, and d_{FRET}^{min} is the minimal distance for an efficient FRET-quenched emission. If a set of disjoint geometric subspaces $\{R_{AB}^i\}$ fulfills the criteria of a bound state, the ensemble of the bound set is the union of these subsets,

$$R_{AB} = R_{AB}^1 \cup R_{AB}^2 \cup R_{AB}^3 \cup \ldots \cup R_{AB}^N \tag{21}$$

$$R_{AB}^i \cap R_{AB}^j = \emptyset \quad \text{for all} \quad i \neq j. \tag{22}$$

The bound-state free energy is then a combination of these states,

$$\begin{aligned} G_{AB} &= G_{r_{sol} \in R_{AB}} \\ &= -\beta^{-1} \ln \sum_i \int \int e^{-\beta U(r, r_{wat})} \chi_{R_{AB}^i}(r) dr dr_{wat} \\ &= -\beta^{-1} \ln \sum_i Z_{AB}^i \\ &= -\beta^{-1} \exp[\sum_i \ln(-\beta G_{AB}^i)], \end{aligned} \tag{23}$$

where Z_{AB}^i is the partition function of each bound state configuration R_{AB}^i with an associated free energy G_{AB}^i. If one bound configuration R_{AB}^* is energetically more favorable than the other states, i.e., $Z_{AB}^* >> \sum_{i \neq *} Z_{AB}^i$, the bound-state free energy is dominated by that state,

$$G_{AB} \approx G_{AB}^*. \tag{24}$$

On the contrary, the unbound-state free energy only accounts for the configurations with $d_{FRET} > d_{FRET}^{min}$ where the interaction between the two proteins becomes negligible

$$\begin{aligned} G_{unbound} &= G_{r \notin R_{AB}} \\ &\approx -\beta^{-1} \ln \int \int \int e^{-\beta U(r_A, r_{wat})} e^{-\beta U(r_B, r_{wat})} dr_A dr_B dr_{wat} \\ &= G_A + G_B. \end{aligned} \tag{25}$$

From Eqs. 24 and 25, the binding free energy to a predominant bound state is obtained,

$$\Delta G_{bind} \approx \Delta G^{*}_{bind} = G^{*}_{AB} - (G_A + G_B). \tag{26}$$

However, in protein-protein stabilization, the protein partners might have only weak interaction without the stabilizer. The stabilizing ligand does not always bring the binary complex to its energetically favorable state. Instead, the ligand induces the protein partners to the stabilized bound state $R^s_{AB} = R^s_A \times R^s_B \times R^s_{A-B}$, with an associated free energy G^s_{AB}

$$G^s_{AB} = G_{r \in R^s_{AB}} = -\beta^{-1} \ln \int\int e^{-\beta U(r_{sol}, r_{wat})} \chi_{R^s_{AB}}(r_{sol}) dr_{wat} dr_{sol}. \tag{27}$$

In practice, the indicator function $\chi_{R^s_{AB}}(r_{sol})$ can be described by an infinitesimal probability cutoff ϵ in the protein-ligand-protein ensemble, namely

$$\chi_{R^s_{AB}}(r_{sol}) = \begin{cases} 1, & \text{if } P_{ALB}(r_{sol}) > \epsilon \\ 0, & \text{otherwise} \end{cases} \tag{28}$$

$$P_{ALB}(r_{sol}) = \frac{\int e^{-\beta U(r_A, r_B, r_L, r_{wat})} dr_{wat} dr_L}{Z_{ALB}}, \tag{29}$$

where $P_{ALB}(r)$ is the probability of the molecules A and B in configuration r_{sol} with the stabilizing ligand in configuration r_L, and Z_{ALB} is the partition function that normalizes probability. Consider the stabilized unary states R^s_A and R^s_B, which fulfill the indicator function of the individual proteins $\chi_{R^s_A}(r_A)$ and $\chi_{R^s_B}(r_B)$, the corresponding free energies of the stabilized unary states are,

$$G^s_A = -\beta^{-1} \ln \int\int e^{-\beta U(r_A, r_{wat})} \chi_{R^s_A}(r) dr_A dr_{wat} \tag{30}$$

$$G^s_B = -\beta^{-1} \ln \int\int e^{-\beta U(r_B, r_{wat})} \chi_{R^s_B}(r) dr_B dr_{wat}. \tag{31}$$

The binding free energy to the stabilized state can be formulated as,

$$\begin{aligned} \Delta G^s_{bind} &\approx G^s_{AB} - (G_A + G_B) \\ &= (G^s_A - G_A) + (G^s_B - G_B) + (G^s_{AB} - G^s_A - G^s_B) \\ &= \Delta G^s_{geo,A} + \Delta G^s_{geo,B} + \Delta G^s_{geo,A-B}, \end{aligned} \tag{32}$$

where $\Delta G^s_{geo,A}$ and $\Delta G^s_{geo,B}$ are the geometric free energy cost, sometimes called reorganization energy in the literature, to bring the proteins to the stabilized subspace R^s_A and R^s_B. $\Delta G^s_{geo,A-B}$ is the geometric free energy difference to bring the pre-organized proteins A and B to the stabilized binary state R^s_{AB}. Note that $\Delta G^s_{geo,A}$, $\Delta G^s_{geo,B}$ are always positive,

$$\Delta G^s_{geo,A} = G_A - G^s_A = -\beta^{-1} \ln \int\int e^{-\beta U(r_A, r_{wat})} [1 - \chi_{R^s_A}(r)] dr_A dr_{wat} \tag{33}$$

$$\Delta G^s_{geo,B} = G_B - G^s_B = -\beta^{-1} \ln \int\int e^{-\beta U(r_B, r_{wat})} [1 - \chi_{R^s_B}(r)] dr_B dr_{wat}. \tag{34}$$

Because $R^s_{AB} \subseteq R_{AB}$, the free-energy difference between the complete bound state and the stabilized bound state is also always positive,

$$\begin{aligned}\Delta G^s_{geo,AB} &= G_{AB} - G^s_{AB} \\ &= -\beta^{-1} \ln \int\int e^{-\beta U(r,r_{wat})} [\chi_{R_{AB}}(r) - \chi_{R^s_{AB}}(r)] dr dr_{wat}.\end{aligned} \tag{35}$$

Substituting Eq. 35 into Eq. 32 gives rise to

$$\Delta G^s_{geo,AB} - \Delta G^s_{geo,A} - \Delta G^s_{geo,B} = \Delta G^s_{geo,A-B} - \Delta G_{bind}, \tag{36}$$

of which the thermodynamic cycle is depicted in Figure S1. In the main text, $\Delta G^s_{geo,A-B}$ is renamed to $\Delta G^{induced}_{A-B}$ to relate its physical meaning that quantifies the intermolecular contribution between the stabilized proteins R^s_A and R^s_B in the stabilized binary complex R^s_{AB}.

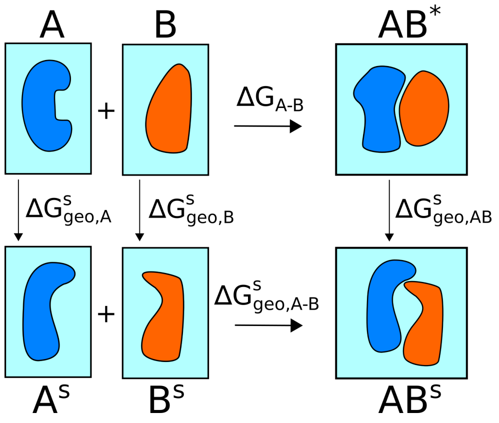

Figure S2: Thermodynamic cycle of the solute geometric change. Note that $\Delta G^s_{geo,A-B}$ is renamed to $\Delta G^{induced}_{A-B}$ in the main text.

S3 Semi-analytical Model for the Estimation of the Cooperative Solvation Contribution

To obtain a more quantitative understanding of the cooperative solvation contribution, empirical methods can be used to decompose $\Delta\Delta G_{\phi,solv}$ into the polar and the apolar terms,

$$\Delta\Delta G_{\phi,solv} = \Delta\Delta G_{\phi,pol} + \Delta\Delta G_{\phi,apol}. \tag{37}$$

The polar part of the solvation-free energy of a set of atoms with interatomic distances r_{ij} and charges q_i can be described by the pairwise semi-analytical generalized-Born (GB) model,

$$\Delta G_{solv,pol} = -\frac{1}{2}(\frac{1}{\epsilon_{in}} - \frac{1}{\epsilon_{sol}}) \sum_{i,j \in solute} \langle \frac{q_i q_j}{f_{ij}} \rangle \tag{38}$$

$$f_{ij}(r_{ij}, R_i, R_j) = \sqrt{r^2_{ij} + R_i R_j \exp(\frac{r^2_{ij}}{4R_i R_j})}. \tag{39}$$

Here, ϵ_{in} and ϵ_{sol} are the dielectric constants of the protein interior and the solvent environment, R_i is the effective Born radii describing the solvent shielding effect from the neighboring atoms, index i and j loop through all solute atoms in the system, and the angular brackets indicate the ensemble average. By reformulating the equation of the polar part (Eq. 37) into a pairwise GB sum of the intramolecular and intermolecular contributions, one obtains,

$$\Delta\Delta G_{\phi,pol} = \sum_{X=A,B,L} \Delta\Delta G^{X}_{\phi,pol} + \sum_{XY=AB,AL,BL} \Delta\Delta G^{XY}_{\phi,pol}. \tag{40}$$

Here, $\Delta\Delta G^{X}_{\phi,pol}$ describes how the engagement of the third party changes how molecule X interacts with the solvent molecules, compared to the binary form, and $\Delta\Delta G^{XY}_{\phi,pol}$ describes how the engagement of the third party changes how the molecule X alters the solvation state of molecule Y. For example,

$$\Delta\Delta G^{A}_{\phi,pol} = -\frac{1}{2}(\frac{1}{\epsilon_{in}} - \frac{1}{\epsilon_{sol}}) \sum_{i\in\{A\}} \sum_{j\neq i\in\{A\}} ((\langle\frac{q_i q_j}{f_{ij}}\rangle_{ALB} + \langle\frac{q_i q_j}{f_{ij}}\rangle_{A} - \langle\frac{q_i q_j}{f_{ij}}\rangle_{AL} - \langle\frac{q_i q_j}{f_{ij}}\rangle_{AB}) \tag{41}$$

$$\Delta\Delta G^{AB}_{\phi,pol} = -\frac{1}{2}(\frac{1}{\epsilon_{in}} - \frac{1}{\epsilon_{sol}}) \sum_{i\in\{A\}} \sum_{j\in\{B\}} ((\langle\frac{q_i q_j}{f_{ij}}\rangle_{ALB} - \langle\frac{q_i q_j}{f_{ij}}\rangle_{AB}) \tag{42}$$

In a static conformation with a fixed-charge description, Eqs. 41 and 42 characterize the difference of the effective Born radii in different complexation states. Figure S3A depicts how the Born radii change on the four atoms located at the A-solvent interface (atom i), binary A-L interface (atom j), binary A-B interface (atom k), and ternary interface (atom m) in the four different complexation states, showing that only the atoms at the three-body interface contribute in Eq. 40.

The apolar term can be estimated by an empirical formula proportional to the difference in solvent-accessible surface area (SASA),

$$\Delta\Delta G_{\phi,apol} = \gamma\Delta\Delta SASA_{\phi} - b \tag{43}$$

where γ is the surface tension, and b is the correction constant (γ=0.005 kcal$\cdot$ mol$^{-1} \cdot \mathring{A}^{-2}$ and b = 0 in the AMBER GB5 model). The net change in SASA during the reduced cooperative process can be also split into the unary and binary parts,

$$\Delta\Delta SASA_{\phi} = \sum_{X=A,B,L} \Delta\Delta SASA^{X}_{\phi} + \sum_{XY=AB,AL,BL} \Delta\Delta SASA^{XY}_{\phi}, \tag{44}$$

where

$$\Delta\Delta SASA^{A}_{alpha_0} = \langle SASA^{A}\rangle_{ALB} + \langle SASA^{A}\rangle_{A} - \langle SASA^{A}\rangle_{AL} - \langle SASA^{A}\rangle_{AB} \tag{45}$$

$$\Delta\Delta SASA^{AB}_{\phi} = \langle SASA^{AB}\rangle_{ALB} - \langle SASA^{AB}\rangle_{AB} \tag{46}$$

Here, $SASA^{X}$ is the SASA of the molecule $X \in \{A, B, L, AB, AL, BL, ALB\}$. In a static configuration, $\Delta SASA_{\phi}$ captures the SASA of the triplet intersection depicted in Figure S3B.

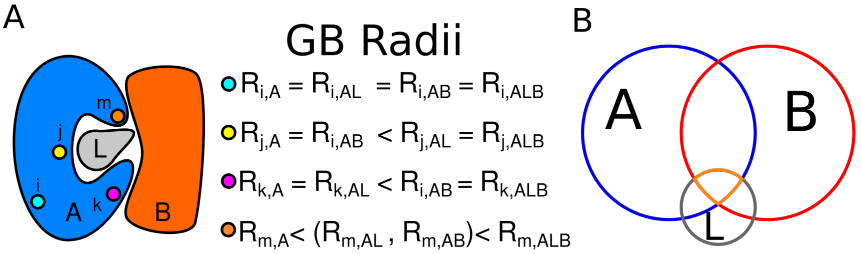

Figure S3: Estimation of the source of the cooperative solvation contribution in ternary complexation. (**A**): Generalized Born (GB) radii of the atoms on protein A at the solvent-exposed surface (cyan), A-L interface (yellow), and A-B interface (magenta). Comparison of the GB radii in the unary, binary, and ternary complex estimated by the closest distance to the solvent-accessible surface. (**B**): Schematic illustration of the $\Delta SASA^s_\phi$ (orange line) at the three-body interface between protein A (blue line), protein B (red line), and ligand L (gray line).

S4 Estimation of the Gas-phase Contribution to the Reduced Cooperativity

The gas-phase reduced cooperative free energy in the reduced cooperative free energy can be reformulated as the difference between gas-phase binding free energies of the ligand to the protein-protein binary complex and to the individual proteins,

$$\begin{aligned}\Delta G^s_{\phi,gas} &= G^s_{ALB,gas} - (G^s_{AL,gas} + G^s_{BL,gas} + G^s_{AB,gas}) + (G^s_{A,gas} + G^s_{B,gas} + G^s_{L,gas}) \\ &= \Delta G^s_{AB-L,gas} - \Delta G^s_{A-L,gas} - \Delta G^s_{B-L,gas}.\end{aligned} \tag{47}$$

By rewriting the binary gas-phase binding free energy in an ensemble-reweighting form

$$\begin{aligned}\Delta G^s_{A-L,gas} &= G_{AL^s,gas} - (G_{A^s,gas} + G_{L^s,gas}) \\ &= -\beta^{-1}\frac{\int\int e^{-\beta(U_A+U_L+U_{A-L})}dr^s_A dr^s_L}{\int\int e^{\beta U_{A-L}} e^{-\beta(U_A+U_L+U_{A-L})}dr^s_A dr^s_L} \\ &= \beta^{-1}\ln\langle e^{\beta U_{A-L}}\rangle_{AL^s,gas},\end{aligned} \tag{48}$$

where U_A and U_L is the intramolecular potential energy of protein A and ligand L, and U_{A-L} is interaction energy between them. Eq. 47 can be expressed in the form of a cumulant-generating function,

$$\begin{aligned}\Delta G^s_{\phi,gas} &= \beta^{-1}(\ln\langle e^{\beta(U_{A-L}+U_{B-L})}\rangle_{ALB^s,gas} - \ln\langle e^{\beta U_{A-L}}\rangle_{AL^s,gas} - \ln\langle e^{\beta U_{B-L}}\rangle_{BL^s,gas}) \\ &= \sum_{n=1}^{\infty}\frac{\beta^{n-1}}{n!}[\kappa^{ALB^s}_n(U_{A-L}+U_{B-L}) - \kappa^{AL^s}_n(U_{A-L}) - \kappa^{BL^s}_n(U_{B-L})],\end{aligned} \tag{49}$$

where $\kappa^X_n(U)$ is n^{th} the cumulant of the interaction energy U in ensemble X. When the two intermolecular interactions U_{A-L} and U_{B-L} are uncorrelated, the joint cumulant becomes cumulative, namely $\kappa^{ALB^s}_n(U_{A-L}+U_{B-L}) = \kappa^{ALB^s}_n(U_{A-L}) + \kappa^{ALB^s}_n(U_{B-L})$. In addition, if the ensembles in the stabilized configurational space are highly overlapped so that $\kappa^{ALB^s}_n(U_{A-L}) \approx \kappa^{AL^s}_n(U_{A-L})$ and $\kappa^{ALB^s}_n(U_{B-L}) \approx \kappa^{BL^s}_n(U_{B-L})$, the gas-phase reduced cooperative free energy becomes negligible for

two uncorrelated ligand binders,

$$\begin{aligned}\Delta G^{s,uncorr}_{\phi,gas} &= \sum_{n=1}^{\infty} \frac{\beta^{n-1}}{n!}[\kappa_n^{ALB^s}(U_{A-L}) + \kappa_n^{ALB^s}(U_{B-L}) - \kappa_n^{AL^s}(U_{A-L}) - \kappa_n^{BL^s}(U_{B-L})] \\ &\approx 0.\end{aligned} \tag{50}$$

Essentially, $\Delta G^s_{\phi,gas}$ in Eq. 49 quantifies the difference in the interaction entropy [1] between the two independent binding events and one simultaneous binding event. Although the interaction entropies in the strongly interacting systems are difficult to compute [2], their difference becomes negligible when two binding events are independent.

S5 Symmetric Description of Cooperativity

Because of the asymmetric nature of the cooperativity (of the ligand) typically used in the literature, the mathematical derivations in cooperative free energy are forced to be also in an asymmetric form. Starting from what is derived in the main text, the effective three-body binding free energy can be written as the sum of the three pairwise binding free energies and the reduced cooperative free energy,

$$\begin{aligned}\Delta G_3 &= \beta^{-1} \log \frac{K_3}{c_0^2} \\ &= \beta^{-1} \log \left(\frac{c_0}{\phi} \frac{K_{A-B}K_{A-L}K_{B-L}}{c_0^3}\right) \\ &= \Delta G_\phi + \Delta G_{A-B} + \Delta G_{A-L} + \Delta G_{B-L},\end{aligned} \tag{51}$$

where the reduced cooperative free energy ΔG_ϕ has the form,

$$\Delta G_\phi = \Delta\Delta G^s_{\phi,geo} + \Delta\Delta G^s_{\phi,solv} + \Delta G^s_{\phi,gas}. \tag{52}$$

$$\Delta\Delta G^s_{\phi,geo} = -\Delta G^s_{geo,ALB} + (\Delta G^s_{geo,AL} + \Delta G^s_{geo,BL} + \Delta G^s_{geo,AB}) - (\Delta G^s_{geo,A} + \Delta G^s_{geo,B} + \Delta G^s_{geo,L}) \tag{53}$$

$$\Delta\Delta G^s_{\phi,solv} = \Delta G^s_{solv,ALB} - (\Delta G^s_{solv,AL} + \Delta G^s_{solv,BL} + \Delta G^s_{solv,AB}) + (\Delta G^s_{solv,A} + \Delta G^s_{solv,B} + \Delta G^s_{solv,L}) \tag{54}$$

$$\Delta G^s_{\phi,gas} = G^s_{ALB,gas} - (G^s_{AL,gas} + G^s_{BL,gas} + G^s_{AB,gas}) + (G^s_{A,gas} + G^s_{B,gas} + G^s_{L,gas}). \tag{55}$$

We can reformulate all binary perturbations as the free difference between the stabilized binary complex and the energetically minimal binary complex, as done in the main text for AB

$$\Delta G^s_{geo,AB} = G^s_{AB} - G_{AB} = \Delta G^{induced}_{A-B} - \Delta G_{A-B} + \Delta G^s_{geo,A} + \Delta G^s_{geo,B}. \tag{56}$$

$$\Delta G^s_{geo,AL} = G^s_{AL} - G_{AL} = \Delta G^{induced}_{A-L} - \Delta G_{A-L} + \Delta G^s_{geo,A} + \Delta G^s_{geo,L}. \tag{57}$$

$$\Delta G^s_{geo,BL} = G^s_{BL} - G_{BL} = \Delta G^{induced}_{B-L} - \Delta G_{B-L} + \Delta G^s_{geo,B} + \Delta G^s_{geo,L}. \tag{58}$$

Therefore, the geometric part can be reformulated into

$$\begin{aligned}\Delta\Delta G^s_{\phi,geo} =& (\Delta G^{induced}_{A-B} + \Delta G^{induced}_{A-L} + \Delta G^{induced}_{B-L} - \Delta G_{A-B} - \Delta G_{A-L} - \Delta G_{B-L}) \\ & - (\Delta G^s_{geo,A} + \Delta G^s_{geo,B} + \Delta G^s_{geo,L} + \Delta G^s_{geo,ALB}).\end{aligned} \tag{59}$$

The cooperative solvation part can be written as the sum of the polar and the apolar contribution noted in Eqs. 40 and 44, ignoring the correction constant b, so that

$$\Delta\Delta G^s_{\phi,solv} = \sum_{X=A,B,L} (\Delta\Delta G^X_{\phi,pol} + \gamma\Delta\Delta SASA^X_\phi) + \sum_{XY=AB,AL,BL} (\Delta\Delta G^{XY}_{\phi,pol} + \gamma\Delta\Delta SASA^{XY}_\phi). \tag{60}$$

Collecting all binary terms in Eqs. 59 and 60 together, we obtain the binary cooperative terms defined by α_0, β_0, and γ_0

$$\Delta G_{\alpha_0} = \Delta G^{induced}_{A-B} + (\Delta\Delta G^{AB}_{\phi,pol} + \gamma\Delta\Delta SASA^{AB}_\phi) - \Delta G_{A-B} \tag{61}$$

$$\Delta G_{\beta_0} = \Delta G^{induced}_{A-L} + (\Delta\Delta G^{AL}_{\phi,pol} + \gamma\Delta\Delta SASA^{AL}_\phi) - \Delta G_{A-L} \tag{62}$$

$$\Delta G_{\gamma_0} = \Delta G^{induced}_{B-L} + (\Delta\Delta G^{BL}_{\phi,pol} + \gamma\Delta\Delta SASA^{BL}_\phi) - \Delta G_{B-L}. \tag{63}$$

We used ϕ_0 to describe the unary and ternary perturbations and correlation effect in the three-body dynamics that are left with the reduced cooperative free energy,

$$\begin{aligned}\Delta G_{\phi_0} &= \Delta G_\phi - \Delta G_{\alpha_0} - \Delta G_{\beta_0} - \Delta G_{\gamma_0} \\ &= \sum_{X=A,B,L} (\Delta\Delta G^X_{\phi,pol} + \gamma\Delta\Delta SASA^X_\phi - \Delta G^s_{geo,X}) + (\Delta G^s_{\phi,gas} - \Delta G^s_{geo,ALB}).\end{aligned} \tag{64}$$

Substituting Eqs. 61-64 in Eq. 51, the effective three-body binding free energy can be reformulated as

$$\Delta G_3 = \Delta G_{\phi_0} + (\Delta G_{\alpha_0} + \Delta G_{A-B}) + (\Delta G_{\beta_0} + \Delta G_{A-L}) + (\Delta G_{\gamma_0} + \Delta G_{B-L}). \tag{65}$$

The effective pairwise binding free energy ΔG^{eff}_{A-B} and its effective dissociation constant K^{eff}_{A-B} in the three-body system can be defined as,

$$\begin{aligned}\Delta G^{eff}_{A-B} &= \Delta G_{\alpha_0} + \Delta G_{A-B} \\ &= \Delta G^{induced}_{A-B} + (\Delta\Delta G^{AB}_{\phi,pol} + \gamma\Delta\Delta SASA^{AB}_\phi) \\ &= \beta^{-1}\log K^{eff}_{A-B}.\end{aligned} \tag{66}$$

The difference between ΔG^{eff}_{A-B} and $\Delta G^{induced}_{A-B}$ lies in the solvation perturbation introduced by the third party during the binary stabilization process, as depicted in Figure S4. Note that solvation perturbation can be both positive and negative, unlike unary and binary perturbations. Finally, the effective three-body dissociation constant can be written as

$$\begin{aligned}K_3 =& \frac{[ALB]}{[A][B][L]} \\ =& \phi_0^{-1} K^{eff}_{A-B} K^{eff}_{A-L} K^{eff}_{B-L}.\end{aligned} \tag{67}$$

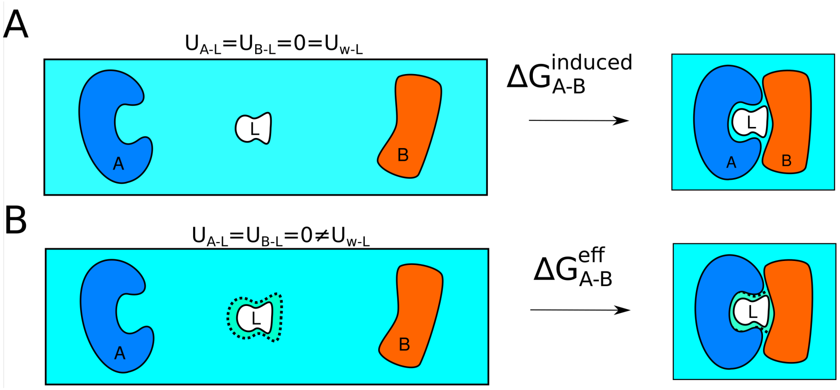

Figure S4: Difference between the induced PPIs and the effective PPIs in the ternary complexes. (**A**): Induced PPIs only consider the binding between the proteins A and B when the ligand does not interact with the solvent ($U_{w-L} = 0$). (**B**): Effective PPIs consider also the perturbed solvent configuration caused by the ligand ($U_{w-L} \neq 0$), highlighted inside the dashed line, previous to the binary complex formation. Proteins A and B are rigid bodies in these examples and do not interact with the ligand ($U_{A-L} = U_{B-L} = 0$).

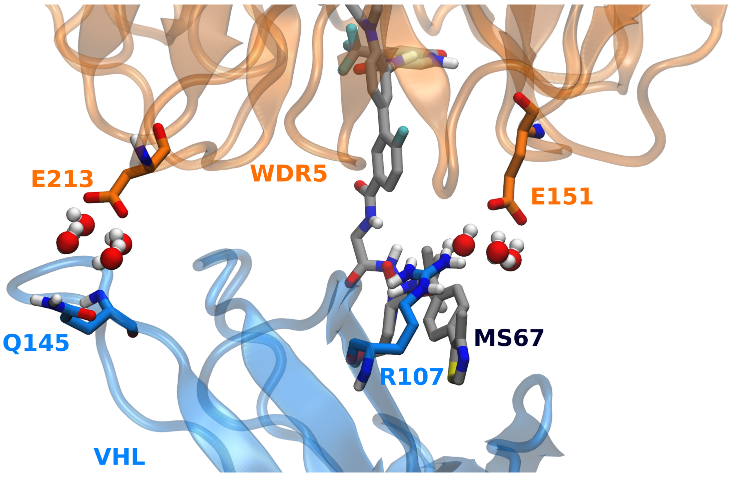

Figure S5: Water-rich protein-protein interface in the VHL(blue)-MS67(grey)-WDR5(orange) complex. Snapshot is taken from the last frame of the first (out of ten) 10 ns simulation.

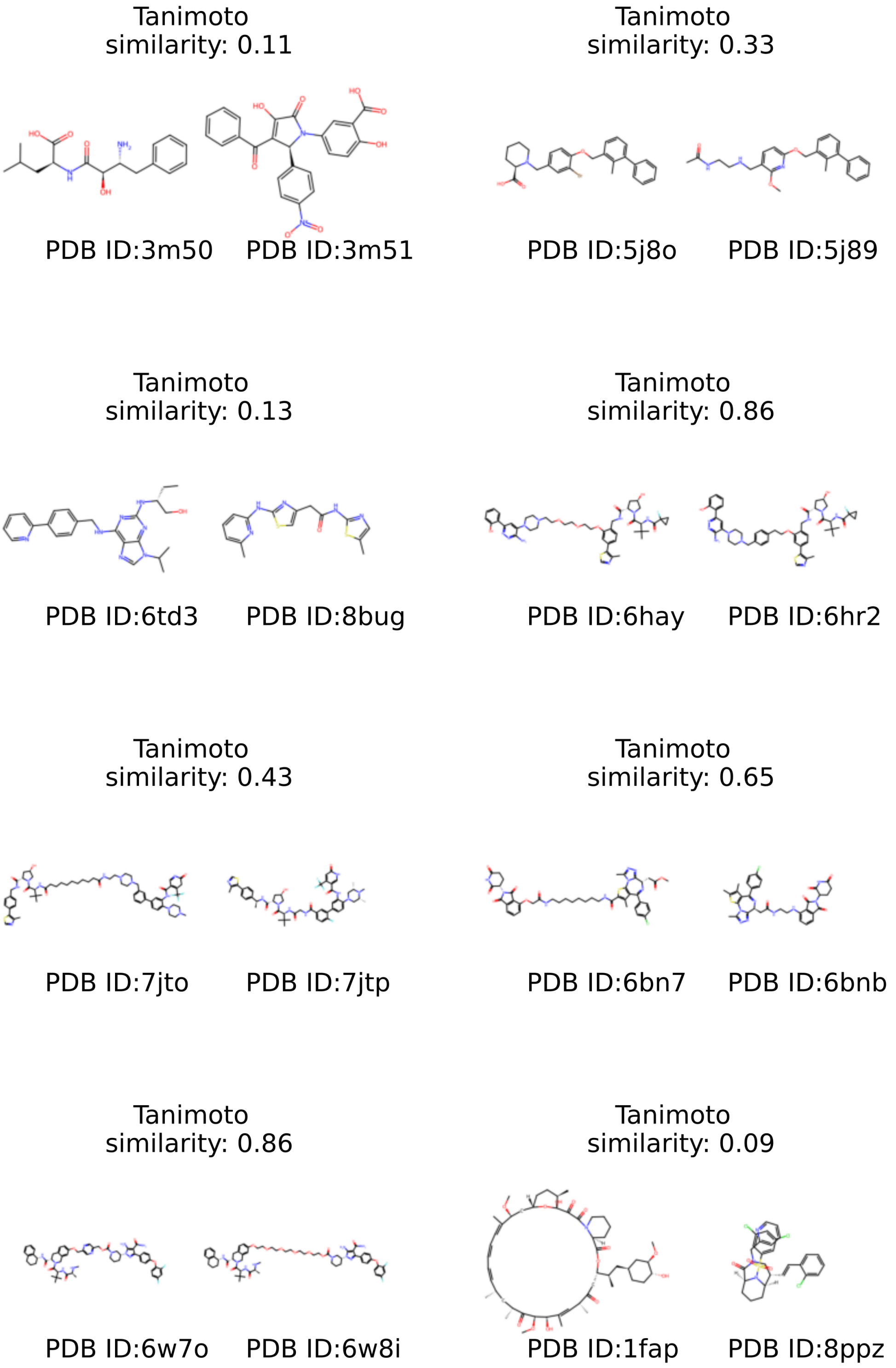

Figure S6: Chemical structure of the examples used in the Figure 5 in the main text. Ligands are drawn and the Tanimoto similarity is calculated with Tanimoto similarity with Morgan fingerprints[3] (2048 bits with a radius of 2) using the RDKit [4].

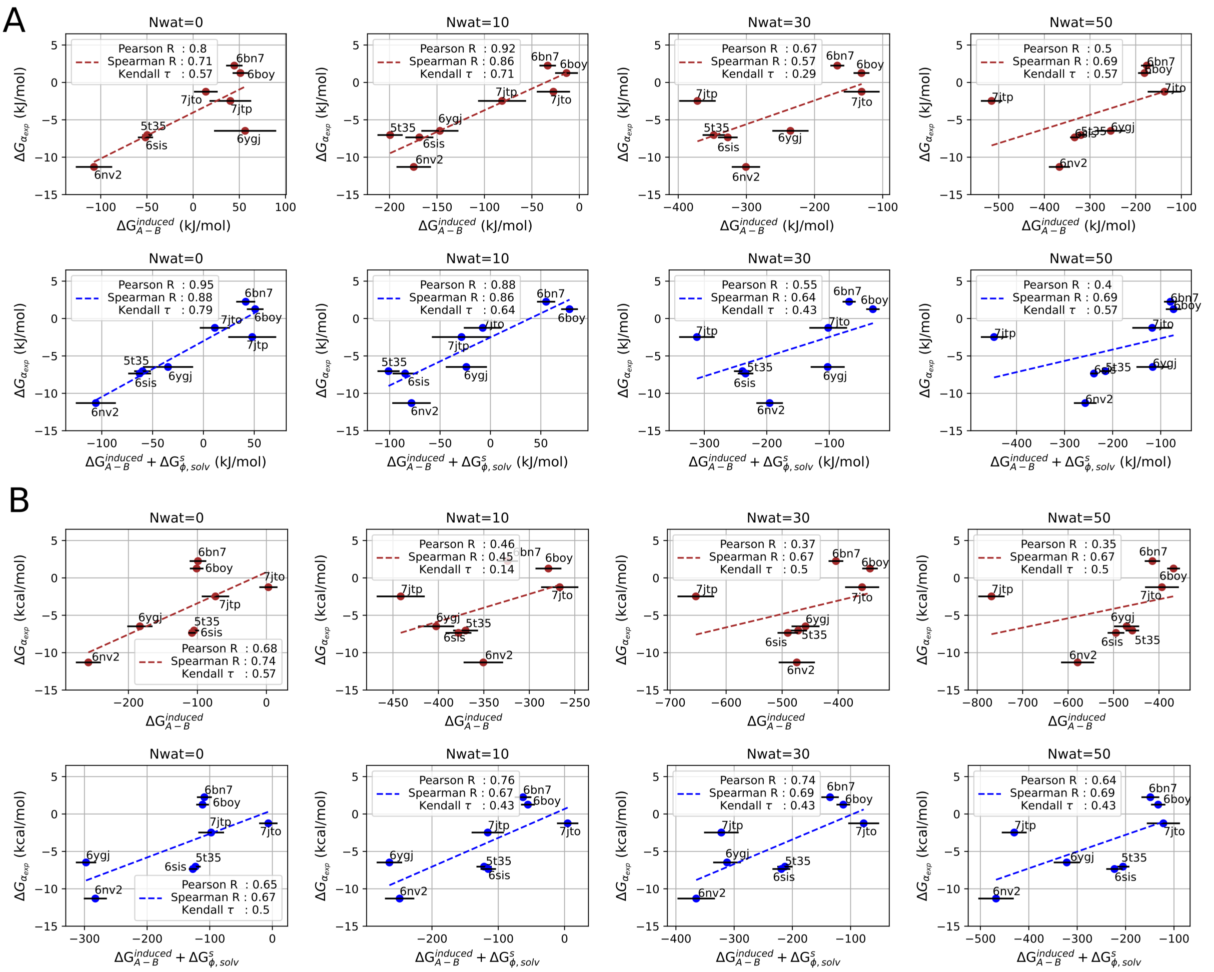

Figure S7: Other endpoint evaluation methods by including N interface water molecules in (**A**) MM/PBSA and (**B**) MM/GBSA. Cooperative free energies estimated with and without the cooperative solvation are shown in blue in the lower rows and brown in the upper rows, respectively.

References

[1] Duan, L.; Liu, X.; Zhang, J. Z. Interaction Entropy: A New Paradigm for Highly Efficient and Reliable Computation of Protein–Ligand Binding Free Energy. *Journal of the American Chemical Society* **2016**, *138*, 5722–5728.

[2] Ekberg, V.; Ryde, U. On the Use of Interaction Entropy and Related Methods to Estimate Binding Entropies. *Journal of Chemical Theory and Computation 17*, 5379–5391.

[3] Rogers, D.; Hahn, M. Extended-Connectivity Fingerprints. *J. Chem. Inf. Model.* **2010**, *50*, 742–754.

[4] Landrum, G. RDKit: Open-Source Cheminformatics Software. **2016**,
